# Identification of diagnostic markers and molecular clusters of cuproptosis-related genes in alcohol-related liver disease based on machine learning and experimental validation

**DOI:** 10.1016/j.heliyon.2024.e37612

**Published:** 2024-09-12

**Authors:** Jiangfa Li, Yong Wang, Zhan Wu, Mingbei Zhong, Gangping Feng, Zhipeng Liu, Yonglian Zeng, Zaiwa Wei, Sebastian Mueller, Songqing He, Guoqing Ouyang, Guandou Yuan

**Affiliations:** a Division of Hepatobiliary Surgery, The First Affiliated Hospital of Guangxi Medical University, Nanning, Guangxi 530021, China; b Key Laboratory of Early Prevention and Treatment for Regional High Frequency Tumor (Guangxi Medical University), Ministry of Education, Nanning, Guangxi 530021, China; c Guangxi Key Laboratory of Immunology and Metabolism for Liver Diseases, Nanning, Guangxi 530021, China; d Center for Alcohol Research, University Hospital Heidelberg, Heidelberg, Germany

**Keywords:** Alcohol-related liver disease, Cuproptosis, Diagnostic, Immune infiltration, Machine learning

## Abstract

**Background and aims:**

Alcohol-related liver disease (ALD) is a worldwide burden. Cuproptosis has been shown to play a key role in the development of several diseases. However, the role and mechanisms of cuproptosis in ALD remain unclear.

**Methods:**

The RNA-sequencing data of ALD liver samples were downloaded from the Gene Expression Omnibus (GEO) database. Bioinformatical analyses were performed using the R data package. We then identified key genes through multiple machine learning methods. Immunoinfiltration analyses were used to identify different immune cells in ALD patients and controls. The expression levels of key genes were further verified.

**Results:**

We identified three key cuproptosis-related genes (CRGs) (DPYD, SLC31A1, and DBT) through an in-depth analysis of two GEO datasets, including 28 ALD samples and eight control samples. The area under the curve (AUC) value of these three genes combined in determining ALD was 1.0. In the external datasets, the three key genes had AUC values as high as 1.0 and 0.917, respectively. Nomogram, decision curve, and calibration curve analyses also confirmed these genes’ ability to predict the diagnosis. These three key genes were found to be involved in multiple pathways associated with ALD progression. We confirmed the mRNA expression of these three key genes in mouse ALD liver samples. Regarding immune cell infiltration, the numbers of B cells, CD8 (+) T cells, NK cells, T-helper cells, and Th1 cells were significantly lower in ALD patient samples than in control liver samples. Single sample gene set enrichment analysis (ssGSEA) was then used to estimate the immune microenvironment of different CRG clusters and CRG-related gene clusters. In addition, we calculated CRG scores through principal component analysis (PCA) and selected Sankey plots to represent the correlation between CRG clusters, gene clusters, and CRG scores. Finally, the three key genes were confirmed in mouse ALD liver samples and liver cells treated with ethanol.

**Conclusions:**

We first established a prognostic model for ALD based on 3 CRGs and robust prediction efficacy was confirmed. Our investigation contributes to a comprehensive understanding of the role of cuproptosis in ALD, presenting promising avenues for the exploration of therapeutic strategies.

## Abbreviations

ALD: alcohol-related liver disease
ALT: alanine aminotransferase
AST: aspartate aminotransferase
AUC: area under curve
CC: cellular component
CDF: cumulative distribution function
CI: credibility interval
CRG: cuproptosis-related gene
DCA: decision curve analysis
DBT: Dihydrolipoamide Branched-Chain Transacylase E2
DE-CRG: differential expression of cuproptosis-related gene
DEGs: differential expression genes
DGIdb: drug-gene interaction databases
DPD: dihydropyrimidine dehydrogenase
DSigDB: drug signatures database
GEO: gene expression omnibus
GO: gene ontology
GSEA: gene set enrichment analysis
GSVA: gene set variation analysis
HE: Hematein & Eosin
KEGG: Kyoto Encyclopedia of Genes and Genomes
LA: lipoic acid
LASSO: least absolute shrinkage and selection operator
NIAAA: National Institute on Alcohol Abuse and Alcoholism
PCA: principal Component Analysis
PPI: protein-protein interaction
RF: random forest
ROC: receiver operating characteristics
ssGSEA: single-sample gene set enrichment analysis
SVM-RFE: support vector machine‐recursive feature elimination
TCA: tricarboxylic acid

## 1 Introduction

Alcohol-related liver disease (ALD) is liver damage caused by prolonged heavy alcohol consumption and encompasses a range of diseases, such as alcoholic fatty liver disease, alcoholic hepatitis, cirrhosis, and complications of cirrhosis [[1], [2], [3]]. ALD is one of the leading causes of chronic liver disease worldwide, accounting for 48 % of cirrhosis-related deaths in the United States [4,5]. ALD, with a comparable incidence in China to that in the United States, is emerging as a significant contributor to the overall liver disease burden in China [6].

Copper is an extracellular element responsible for the precise functioning of the bone marrow and central nervous system and acts as a cofactor for many antioxidant enzymes, such as super oxide dismutase; thus, disorders of copper metabolism may cause organ dysfunction [[7], [8], [9], [10]]. Dysregulation of copper is significantly associated with liver disease [11]. Wilson disease is a classic example of copper overload, an autosomal recessive genetic disorder characterized by multiple mutations in the ATP7B gene [12]. Studies have shown a disturbance of copper metabolism in ALD patients [[13], [14], [15]]. Alcohol diet can cause liver damage and decrease the expression of HIF-1alpha, occludin, SOD1, and GPX1 genes. It was also shown that a lack of copper in the diet may exacerbate these changes, and supplementation with copper may improve these conditions [16]. Furthermore, *in vitro* cell experiments have shown that appropriate copper supplementation can promote cell growth and reduce the production of reactive oxygen species (ROS). Previous studies have shown that moderate ALD is closely related to oxidative stress, and copper deficiency will promote dyslipidemia and increase oxidative stress because copper is an essential cofactor of many antioxidant enzymes; thus, insufficient copper may further promote the damaging effects of alcohol [17]. Alcohol metabolism in the liver cells of patients with excessive alcohol consumption produces a large level of ROS, resulting in elevated lipid peroxidation, which disrupts mitochondrial function and causes liver cell death [18]. Cuproptosis is a new form of cell death associated with many diseases, such as non-ALD,Wilson disease, and hepatocellular carcinoma [[19], [20], [21]].

Research focused on uncovering the relationship between cuproptosis and the underlying pathophysiology of ALD is relatively limited. Therefore, we conducted a comprehensive study of the expression, diagnosis, immune correlation, and mechanism of cuproptosis-related genes (CRGs) in ALD is still needed. In this study, we downloaded ALD-related data from the Gene Expression Omnibus (GEO) database and conducted analysis and machine-learning analyses to explore the relationship between CRGs and ALD. We identified differentially expressed genes and the key genes among them and subsequently built and externally validated a prediction model. Finally, we performed immunoinfiltration analyses, assessed related drugs, and investigated associated competing endogenous RNAs (ceRNAs).

## 2 Materials and methods

### 2.1 Patients and datasets

**Fig. 1:**
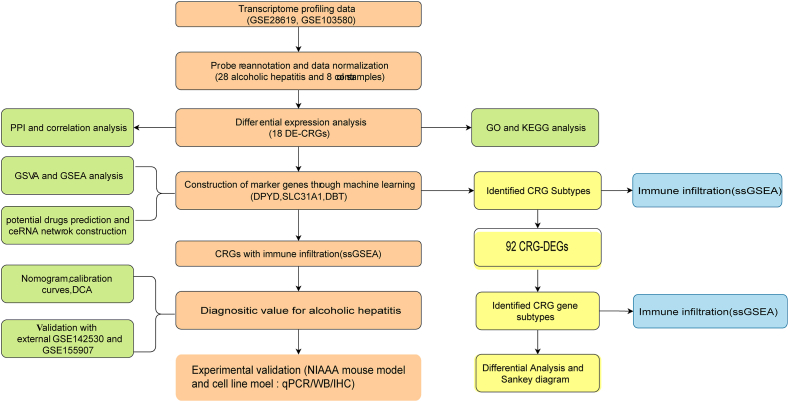
Flowchart of the present study.

The transcriptomic analysis of ALD and normal non-ALD liver specimens included two datasets, GSE28619 and GSE103580, downloaded from the GEO database. Specifically, GSE28619 (based on the GPL570 platform) contained 7 healthy control samples and 15 samples of alcoholic hepatitis. GSE103580 (GPL13667 platform) included 13 cases of alcoholic hepatitis and 1 healthy control. The battle function of the “sva” package was used to eliminate batch effects and potentially unknown variables. After combining data from GSE28619 and GSE103580, the final datasets included 28 cases of alcoholic hepatitis and 8 control cases. The flow chart of this study is shown in Fig. 1.

### 2.2 Expression of differentially expressed genes in ALD

The differential expression analysis was performed using the R package limma based on the GEO database. We identified differentially expressed genes (DEGs) between ALD and healthy samples. The screening criteria were as follows: P < 0.05. The intersection of DEGs related to CRGs was created using the “VennDiagram” R package and defined as DE-CRGs for subsequent analysis.

### 2.3 Correlation analysis and protein-protein interaction network construction

The R software packages “RCircos” and “heatmap” were used to generate landscape maps of 23 chromosomes and heatmaps of 18 DE-CRGs, respectively. The “circlize” software package was used to create correlation Circos plots based on Pearson correlation analysis. Eighteen DE-CRGs protein-protein interaction (PPI) networks were constructed using the Search Tool for the Retrieval of Interacting Genes/Proteins (STRING; https://string-db.org/) database.

### 2.4 Gene ontology and KEGG pathway enrichment analysis

The R package “clusterProfiler” was employed to analyze the biological function of genes through Gene Ontology (GO) and KEGG pathway enrichment analysis. GO annotation included biological processes (BP), cellular components (CC), and molecular functions (MF).

### 2.5 Construction of the CRGs diagnostic model

Two datasets, GSE28619 and GSE103580, were integrated for modeling, and the GSE142530 and GSE155907 datasets were used as external validation sets for verification. Employing the “glmnet” package in R, we conducted the least absolute shrinkage and selection operator (LASSO) regression on the chosen linear model to decrease the data dimension while preserving valuable variables [22,23]. The main idea of recursive feature elimination (REF) was to build the model iteratively and then select the best (or worst) feature (selected according to the coefficient) [24]. The maximum interval principle of the support vector machine (SVM) was the foundation for the sequential reverse selection algorithm known as support vector machine recursive feature elimination (SVM-RFE) [[25], [26], [27]]. We used the “e1071” package in R to screen the best variable genes to establish the SVM-RFE model [28,29]. The random forest (RF) algorithms were combined to select the best genes. The RF model is a regression tree technique that uses bootstrap aggregation and randomization of predictors to achieve high prediction accuracy [30]. The “randomForest” R package was used for RF. The genes selected by three kinds of machine learning were intersected to obtain the final key genes.

### 2.6 Verification of the model

First, we used the “rms” package in R to build a nomogram model to predict the occurrence of ALD. The “pROC” package in R [31] was employed to analyze the area under the curve (AUC), specificity, and sensitivity of diagnostic value for marker genes using a time-dependent ROC. Each central gene was assigned a score, which was then added to form a total score. Two external datasets, GSE142530 and GSE155907, were used to verify the diagnostic capability of the diagnostic model.

### 2.7 Gene set enrichment analysis and gene set variation analysis

Gene set enrichment analysis (GSEA) was conducted using the “clusterProfiler” package to explore the potential functions of the hub genes [32]. In addition, differential expression genes (DEGs) and pathway enrichment analyses were executed with the “gene set variation analysis (GSVA)”, “clusterProfiler”, and “Limma” packages. Statistical significance was determined by enrichment analysis with p < 0.05.

### 2.8 Evaluating immune infiltration

Using the “GSVA” package in R, a single-sample GSEA (ssGSEA) algorithm was employed to assess enrichment scores between various immune cells and functions or pathways between ALD and control groups. We downloaded reference gene collections from a public database (http://www.immport.org). Spearman correlation analysis was used to explore the relationship between the three key genes and the immune score. The Wilcoxon test was used to explore differences in immune cell and immune-related functional enrichment scores. The enrichment scores between the ALD and normal liver sample groups are represented by box plots.

### 2.9 Detection of CRG clusters and gene clusters and their relationship with immune cells

The “ConsensusClusterPlus” package was used to distinguish CRG and Gene clusters according to CRG and gene regulatory factors, respectively. The number of clusters was determined based on a cumulative distribution function (CDF) curve and a specific k value [33]. Then, principal component analysis (PCA) was used to further validate the CRG modification pattern [34]. PCA method was applied to calculate the CRG scores of each sample [35]. The CRG scores of the two clusters were shown in a box graph. Finally, Sankey plots were selected to represent the correlation between CRG clusters, gene clusters, and CRG scores. The ssGSEA was used to estimate the extent of 23 immune cells across different CRG and Gene clusters to explore correlations between them.

### 2.10 Drug-gene interaction analyses and **ceRNA network construction**

Drug-gene interactions were explored using the drug-gene interaction database (DGIdb, https://dgidb.genome.wustl.edu/) [36]. One can forecast the miRNA-mRNA relationship by utilizing the three essential genes in miRDB (http://www.mirdb.org/) and TargetScan (https://www.targetscan.org/vert_80). Evidence of a direct interaction between the miRNA and lncRNA was obtained using SpongeScan (http://spongescan.rc.ufl.edu/). Cytoscape software (version 3.9.0) was utilized to visualize mRNA‒miRNA-lncRNA interactions via the ceRNA network.

### 2.11 ALD mouse model construction and histological procedure

Sixteen 8-week-old female C57BL/6 WT mice were used for this experiment. All mice were administered the Lieber-DeCarli diet for 5 days before feeding to adapt to the liquid diet. The experimental group was then freely administered the Lieber-DeCarli diet containing 5 % (v/v) ethanol for 10 days. The control group was provided with an equal calorie-controlled diet. On day 16, the experimental group was given a single dose of ethanol (6 g/kg body weight, 31.5 % ethanol), and the control group was given isocaloric dextrin-maltose [37]. All mice were euthanized 9 h later, and blood was taken from the inferior vena cava with a 1 ml syringe and centrifuged at 3000 rpm for 15 min. The supernatant was collected to obtain the mouse plasma. Serum aspartate aminotransferase (AST) and alanine aminotransferase (ALT) levels were measured using an automatic biochemical analyzer (ANTECH Diagnostics, Los Angeles, CA, USA). Liver tissue samples were also obtained from the mice. Part of each liver sample was placed in an EP tube and directly frozen in liquid nitrogen. The remaining tissue was fixed in formalin solution, embedded in paraffin, and stained with hematoxylin & eosin (HE) staining. A section of frozen fresh liver tissue was prepared with a thickness of 4 mm and stained with Oil Red O. Rabbit monoclonal antibodies were used for immunohistochemistry: anti-DBT (12451-1-AP), anti-DPYD (27662-1-AP), and anti-SLC31A1 (67221-1-Ig). All animal experiments were approved by the Animal Care and Use Committee of the First Affiliated Hospital of Guangxi Medical University (NO.2023-S606-01).

### 2.12 Cell culture and treatments

The AML12 cell line was supplied by the Cell Bank of Type Culture Collection of the Chinese Academy of Sciences (Shanghai, China). It was cultured in mouse liver parenchymal cell AML12 complete culture medium with DMEM/F-12 (1:1) (Gibco, 11330-032) 89 mL ITS Liquid Media Supplement (Sigmadg, I3146) 1 mL Dexamethasone (Sigma, D4902-100 mg) 40 ng/mL FBS (Gibco) 10 mL at 37μC with 5 % CO_2_ using a cell incubator. When cells reached a confluence of 60–70 %, they were divided into 2 groups (n = 4), including [1] the control group (treated with NS) and [2] the ethanol group (treated with 200 mM ethanol). The treatment duration was 24 h. The dose selection of ethanol was based on a previous study on carbon tetrachloride-induced rat hepatocyte injury model.

### 2.13 Protein extraction and western blotting

Liver samples were homogenized in lysis buffer (Solarbio R0010). Samples were then sonicated and incubated on ice for 30 min. Debris was removed by centrifugation at 12,000 rpm. Cellular total protein was extracted using a M-PER Mammalian Protein Extraction Reagent (Pierce, Rockford, IL). Samples were separated in a denaturing 10 % polyacrylamide gel and transferred to a 0.45 mm pore nitrocellulose membrane. Non-specific binding sites were blocked with 5 % nonfat milk (YAMEI PS112L) for 1 h at room temperature. Membranes were then incubated with appropriate primary antibodies in TBS with 0.1 % Tween 20 (TBST). Membranes were washed and incubated with secondary antibodies conjugated to horseradish peroxidase to show the bands with Omni-ECL buffer (YAMEI SQ201). Parallel blotting of GAPDH (Proteintech 10494-1-AP) was used as an internal control.

### 2.14 Quantitative reverse transcription-polymerase chain reaction (qRT-PCR)

The RNA was extracted from mouse liver tissues and the AML12 cell line with TRIzol reagent (Thermo Fisher Scientific, USA). RNA was extracted and eluted using an RNA binding column, and purified total RNA samples were obtained. The RNA was reverse transcribed using the PrimeScript™ RT reagent Kit (Takara, Japan), and quantitative real-time PCR (qRT-PCR) was performed using the FX Connect system (Bio-Rad, USA) and SYBR® Green Supermix (Bio-Rad, USA). Hub genes expression levels were analyzed using 2−DDCT, and the outcomes were demonstrated using GAPDH as an internal control. To ensure the reliability and comparability of the data, the following normalization criteria were applied:1. We selected GAPDH as reference genes whose expression levels remain constant under different experimental conditions; 2, ΔCt Calculation: The Ct value of the target gene for each sample was normalized to the Ct value of the reference gene, resulting in a ΔCt value; 3. ΔΔCt Method: The ΔCt values of the experimental group were compared with those of the control group to calculate the ΔΔCt value (ΔΔCt = ΔCt (experimental group) - ΔCt (control group)); 4. Calculation of Relative Expression Levels: The relative mRNA expression levels were determined using formula 2^-ΔΔCt. Reverse transcription of the RNA was performed using the PrimeScript™ RT Reagent Kit (Takara, Japan), and qRT-PCR was conducted with an FX Connect system (Bio-Rad, USA) and SYBR® Green Supermix (Bio-Rad, USA). qRT-PCR was repeated three times. The primers used in this study are shown in Supplementary Table S1.

### 2.15 Statistical analyses

Continuous variable data are presented as the mean ± standard deviation. Student's *t*-test was employed to compare two groups, while the Wilcoxon rank-sum test was used to examine non-normally distributed variables. A p value less than 0.05 was considered to indicate a significant difference. The symbols *, **, and *** indicate that the p values were less than 0.05, 0.01, and 0.001, respectively. R software (version 4.2.1) was utilized for all statistical analyses.

## 3 Results

### 3.1 Identification of CRGs involved in ALD

**Fig. 2:**
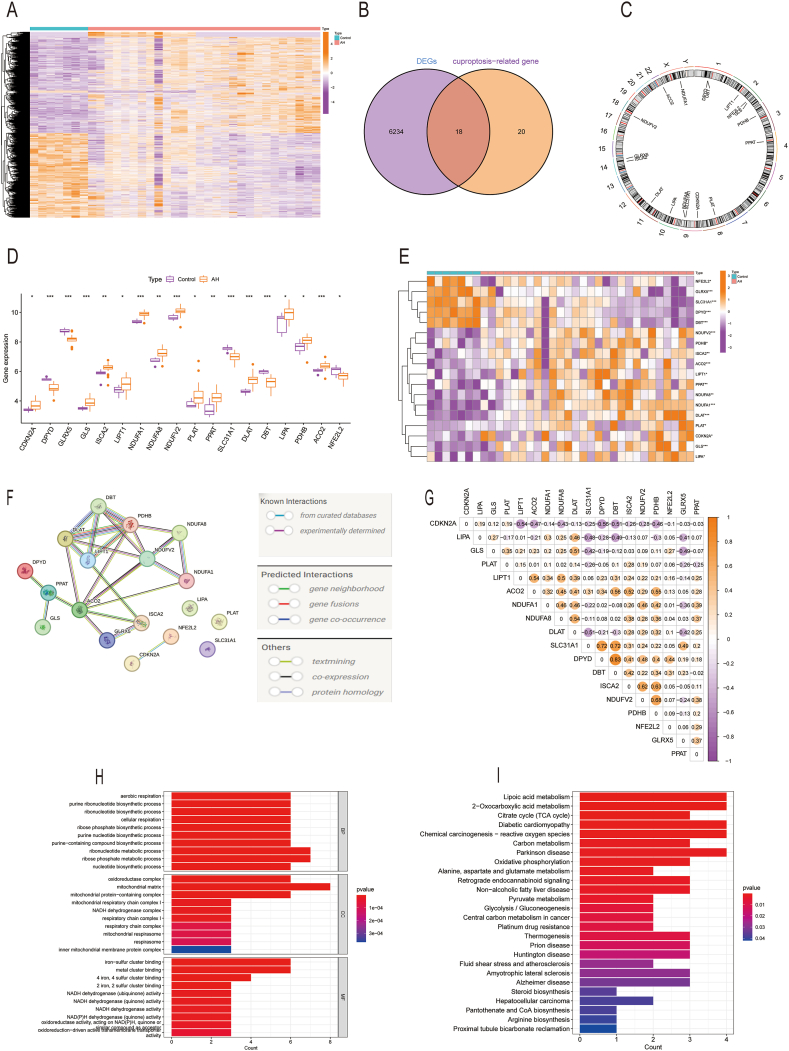
Identification of cuproptosis-related genes in ALD. (A) Heatmap of DEGs was presented. (B) Venn diagrams showing the intersection between DEGs and CRGs. (C) Eighteen DE-CRGs were located on 23 chromosomes, with the boxplots illustrating the differential expression between ALD and control samples (D). (E) Heatmap showed the expression patterns of these 18 DE-CRGs. (F) Gene relationship network diagram of the 18 DE-CRGs. (G) Correlation analysis of the 18 DE-CRGs was conducted, with orange and blue representing positive and negative correlations, respectively. (H) GO analysis of DEGs associated with cuproptosis. (I) KEGG analysis of DEGs associated with cuproptosis. P values are displayed as follows: *p < 0.05; **p < 0.01; ***p < 0.001. ALD, alcohol-related liver disease; CRGs,cuproptosis-related genes; DEGs: Differentially expressed genes. DE-CRGs: differentially expressed cuproptosis-related genes; GO,gene ontology; KEGG, Kyoto Encyclopedia of Genes and Genomes.

A total of 6234 DEGs (p < 0.05) were identified using the “limma” package from two batch-normalized datasets comprising 28 ALD samples and 7 control samples (GSE28619 and GSE103580). Heatmap of DEGs was presented in Fig. 2A. The volcano plot of the DEGs was displayed in Fig. S1, GO analyses of the 6234 DEGs were shown in Fig. S2, and KEGG analyses were shown in Fig. S3.

In addition, 6234 DEGs overlapped with 38 CRGs, revealing 18 DE-CRGs (DLAT, ISCA2, GLRX5, NDUFV2, ACO2, NDUFA1, DPYD, DBT, LIPT1, NFE2L2, GLS, PDHB, PPAT, PLAT, CDKN2A, SLC31A1, NDUFA8, and LIPA) with significant differences between the ALD and control groups, as shown in Fig. 2B. The chromosomal locations of the 18 DE-CRGs were shown in a circular diagram (Fig. 2C). Twelve DE-CRGs (ISCA2, NDUFV2, ACO2, NDUFA1, LIPT1, GLS, PDHB, PPAT, PLAT, CDKN2A, NDUFA8 and LIPA) were upregulated in ALD, and six (DLAT, GLRX5, DPYD, DBT, NFE2L2, and SLC31A1) were downregulated in ALD (Fig. 2D and E). A PPI analysis using STRING was performed to explore potential crosstalk among these 18 DE-CRGs, as shown in Fig. 2F. The correlation among the 18 DE-CRGs was shown in Fig. 2G. We found that DE-CRGs were related to aerobic respiration, purine ribonucleotide biosynthetic, oxidoreductase, and iron-sulfur cluster binding and other pathways with Gene ontology (GO) enrichment analysis including cross gene enrichment in Biological Process (BP), Cellular Component (CC), and Molecular Function (MF). The results were displayed in Fig. 1H. Additionally, lipoic acid (LA) metabolism, citrate cycle, etc., in KEGG pathway analysis was depicted in Fig. 2I.

### 3.2 Identification of diagnostic marker genes for ALD

**Fig. 3:**
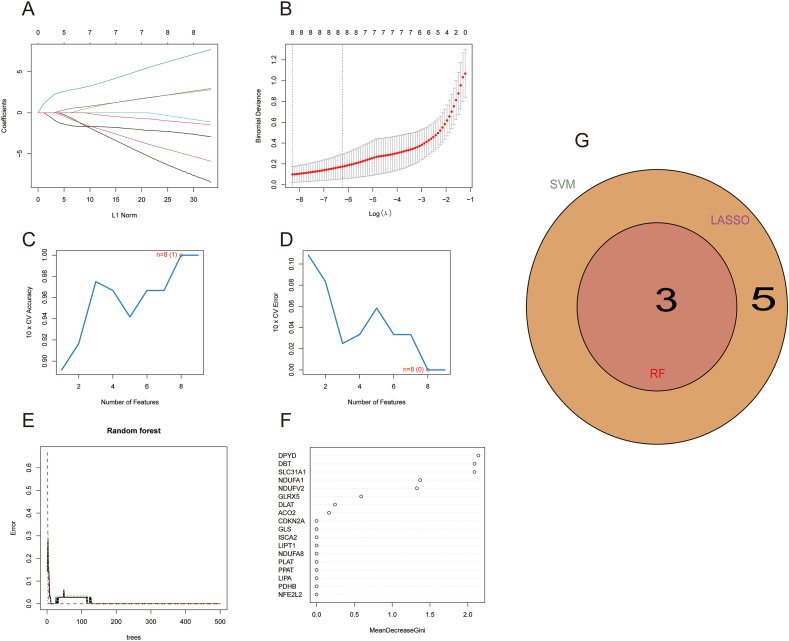
Machine learning was utilized to identify diagnostic marker genes for ALD. (A) The adjusted parameter selection in the LASSO model was cross-validated 10 times, with each curve representing a gene. (B) LASSO coefficient analysis. (C) The SVM-RFE algorithm's feature selection accuracy and error reach a peak of 100 % at eight genes, with the lowest cross-validation error being 0 %. (D) The relationship between random forest tree number and error rate was also evident. (E) The ALD group's error was represented by a red line, the control group's by a green line, and the total sample error by a black line in random forest analysis. (F) Eighteen DG-CRGs were performed with random forest analysis, which three had an average Gini reduction greater than 2. (G) Venn diagram showing overlapping genes obtained using the three machine learning algorithms (LASSO, random forest, and SVM-RFE). ALD, alcohol-related liver disease; LASSO, least absolute shrinkage and selection operator; SVM-RFE, support vector machine‐recursive feature elimination; DE-CRGs, differential expression of cuproptosis-related genes.

Given the individual complexity and heterogeneity of patients with ALD and healthy controls, candidate CRGs were identified from 18 DE-CRGs using LASSO regression and two validated machine learning models (SVM-RFE and RF), which helped predict the diagnosis of ALD. Eight DE-CRGs were identified by the LASSO logistic regression algorithm (Fig. 3A and B). The number of SVM features was 8 (Fig. 3C). The minimum error of the classifier was 0.0, and the maximum accuracy was 1.0 (Fig. 3D). Eighteen DG-CRGs were performed with RF analysis, of which three had an average Gini reduction greater than 2 (Fig. 3E and F). A Venn diagram was then used to intersect the essential genes in the LASSO, SVM-RFE, and RF analyses. Finally, three key genes (DPYD, SLC31A1, and DBT) were identified for further analysis (Fig. 3G).

### 3.3 Evaluation of the diagnostic performance of ALD diagnostic marker genes

**Fig. 4:**
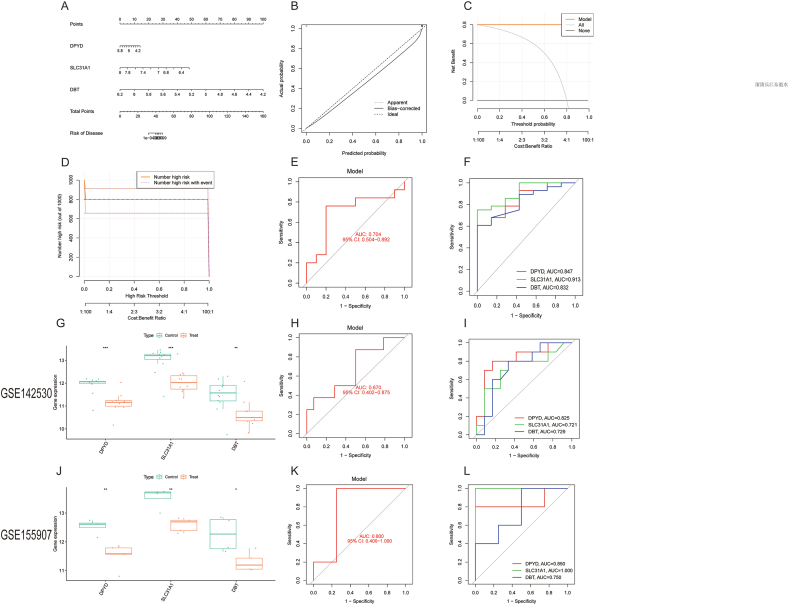
Validation of marker genes expression. (A) Nomogram of marker genes. (B) Calibration curve. (C) The predictive efficiency of the nomogram model was illustrated by DCA. (D) The diagnostic ability of the nomogram model was further demonstrated by the clinical impact curve. (E) The ROC of the combination of the three key genes for the diagnosis of ALD was 0.704 (95 % CI 0.504–0.892). (F) ROC results showed that three marker genes separately diagnosed ALD. The AUC values of DPYD, SLC31A1, and DBT were 0.847, 0.913, and 0.832, respectively. (G) Boxplots of GSE142530 revealed that the three DE-CRGs between the ALD and control samples were significantly different. (H) The three-gene model, based on a threefold cross-validation process in GSE142530, yielded ROC results with an AUC value of 0.0.670. (I) The AUC values of DPYD, SLC31A1, and DBT were 0.825, 0.721, and 0.729, respectively, in GSE142530. (J) The box diagram shows that the three genes differed significantly in different groups of GSE155907 samples. (K) The three-gene model, based on threefold cross-validation in GSE155907, yielded ROC results of 0.800. (L) The AUC values of DPYD, SLC31A1, and DBT for the diagnosis of ALD in GSE155907 were 0.850, 1.0, and 0.750, respectively. DCA, decision curve analysis; ROC, receiver operating characteristics; ALD, alcohol-related liver disease; CI, confidence interval; AUC, area under curve; DE-CRGs, differential expression of cuproptosis-related genes.

A nomogram model of patients with ALD was constructed using the “rms” package to evaluate the predictive efficiency of the three central genes, DPYD, SLC31A1, and DBT (Fig. 4A). The nomogram model's numerical value for each biomarker was used to forecast the ALD risk, with a correction curve indicating an obvious connection between the predicted and actual probability (Fig. 4B). The DCA revealed that the net benefit from this model was significantly higher than 0, implying its remarkable accuracy and ability to provide physicians with a basis for decision-making (Fig. 4C). The clinical impact curve showed that this nomogram model has a very high diagnostic capability (Fig. 4D). The ROC curve analysis showed that the three marker gene features combined demonstrated high performance in diagnosing ALD (AUC = 0.704, Fig. 4E). ROC curves were generated for each of the three labeled genes, and the predictive value of each was assessed. The individual predictive ROC results for these three genes all exceeded 0.83 (Fig. 4F), and their expression was decreased in both datasets (Fig. 4G and J). The ROC curve for the combination of the three genes in the GSE142530 and GSE155907 sets were 0.670 and 0.800, respectively (Fig. 4H and K), and there were the ROC curve for the predicted performance of the three genes separately (Fig. 4I and L). These indications suggest that the model based on these three marker genes may have strong prediction efficacy for ALD.

### 3.4 GSEA, GSVA, and ssGSEA analysis

**Fig. 5:**
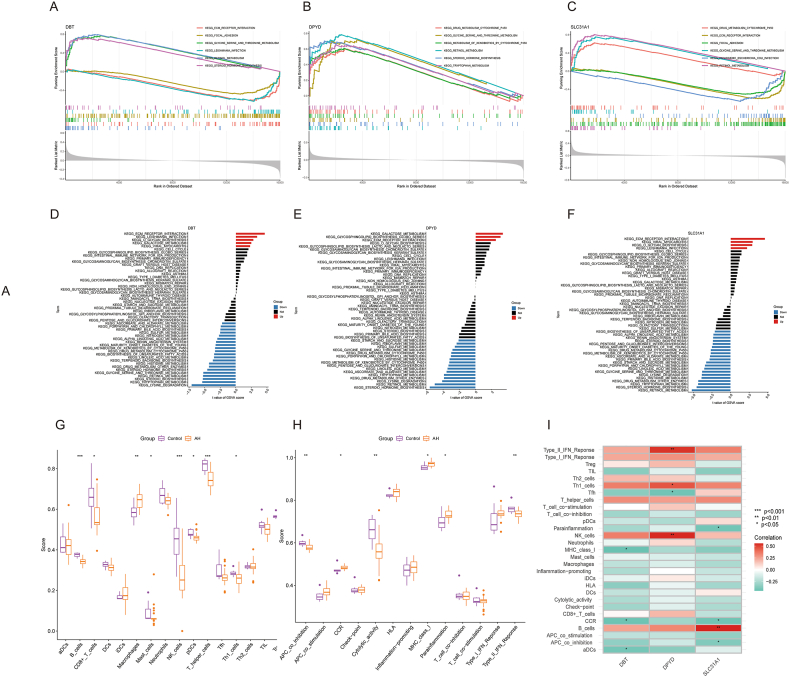
An enrichment analysis of the three marker genes. GSEA analysis of the signature based on (A) ADBT, (B) DPYD, and (C) SLC31A1. KEGG pathway enrichment for (D) DBT, (E) DPYD, and (F) SLC31A1 was performed by GSVA, with the top 50 shown according to the enrichment score. The boxplots demonstrated the disparities in immune cells (G) and function (H) between ALD and non-ALD samples. (I) The correlations of immune cells and three genes were also shown (red: positive correlation, green: negative correlation). p values are indicated as *p < 0.05; **p < 0.01; ***p < 0.001. GSEA, gene set enrichment analysis; GSVA, gene set variation analysis; ALD, alcohol-related liver disease.

We used ssGSEA to identify the major signaling pathways of the three genes in the above model. GSEA of the KEGG pathways demonstrated that these three genes are implicated in glycine, serine, threonine, and retinol metabolism (Fig. 5A–C). DPYD and SLC31A1 are involved in cytochrome p450 metabolism (Fig. 5B and C). DBT and SLC31A1 are related to hormone biosynthesis (Fig. 5A and C). DPYD is involved in tryptophan metabolism, and the influence of cytochrome p450 on genomics (Fig. 5B). Figs. S4–S6 shows the GSEA outcome for the GO enrichment analysis.

GSVA revealed distinct activity pathways between low- and high-expression subtypes determined according to the levels of the three hub genes. Our analysis revealed that DBT, DPYD, and SLC31A1 overexpression are involved in extracellular matrix-receptor interactions (Fig. 5D–F). Overexpression of DBT and SLC31A1 is involved in *Leishmania* infection, glycan biosynthesis, and viral myocarditis. In the high DPYD expression group, galactose metabolism globo-series base biosynthesis pathways were active (Fig. 5E). Low DBT, DPYD, and SLC31A1 expression levels were linked to lysine degradation, tryptophan metabolism, retinol metabolism, threonine metabolism, steroid hormone biosynthesis, and other pathways (Fig. 5D–F). The GSVA result for the GO enrichment analysis is shown in Figs. S7–S9.

In order to verify whether cuproptosis could promote ALD progression by mediating immune infiltration, we conducted ssGSEA analysis. ssGSEA analysis showed that significantly decreased B cells, CD8 (+) T cells, NK cells, T-helper cells, and Th1 cells were observed in ALD patients versus normal liver tissue (Fig. 5G). Regarding immune function, the APC coinhibition, cytolytic activity, and type II IFN response scores were lower in the ALD group than in the control group (Fig. 5H). Fig. 5F shows that DPYD was significantly connected with the type II IFN response, Th1 cells, and NK cells; DBT was significantly associated with MHC class 1, CCR, and aDCs. Whereas, SLC31A1 was significantly correlated with B cells, APC coinhibition, and CCR (Fig. 5I).

### 3.5 The correlation between CRG clusters and the immune microenvironment

**Fig. 6:**
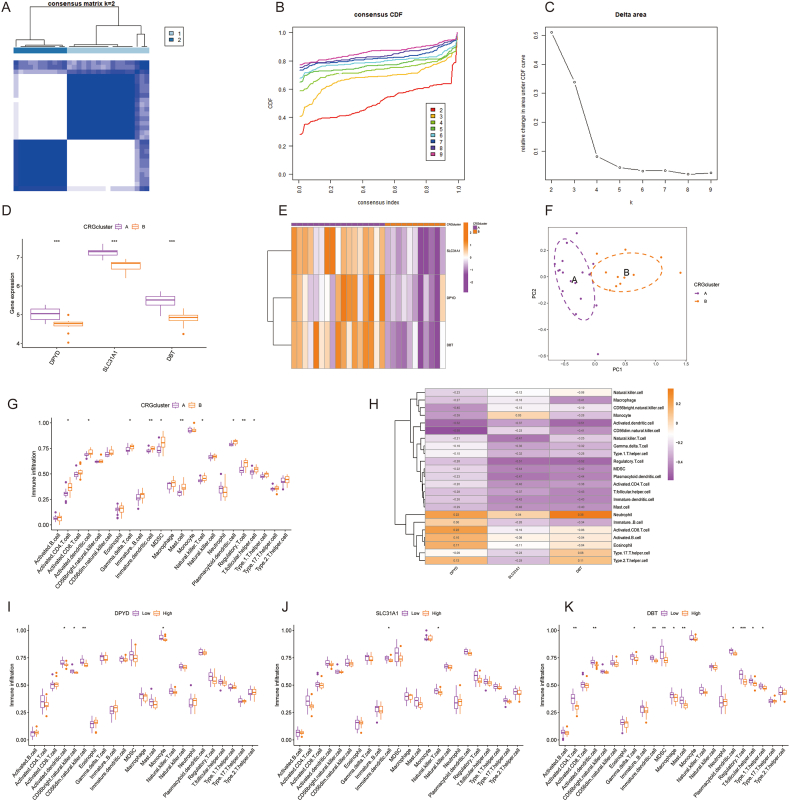
Correlation between CRG clusters and the immune microenvironment. (A) The three CRG hub genes were clustered into CRG clusters A and B under unsupervised consensus clustering (k = 2). (B) The consistent distribution of various k (ranging from 2 to 9) was described using the experience CDF graph. (C) Relative increase in cluster stability by delta area fraction. (D) The boxplots displayed the expression level of three CRG regulator genes between different CRG clusters. (E) The heatmap illustrated the transcriptional profile of CRG regulator genes in each cluster. (F) PCA further revealed a significant difference between CRG clusters A and B. (G) Different cells in immune microenvironment in both groups were presented in the boxplots. (H) Heatmap showed the correlation between CRG regulator genes and the different immune cells. (I–K) The correlations between three key genes and different immune cells. *p < 0.05, **p < 0.01, ***p < 0.001. CRGs, cuproptosis-related genes; CDF, cumulative distribution function; PCA, principal Component Analysis.

The “Consensus Cluster Plus” package in R classified different CRG clusters according to three hub gene regulators. Taking 2 as the optimal k value, 28 ALD samples were divided into CRG clusters A and B (Fig. 6A–C). DBT, DPYD, and SLC31A1 expression levels were higher in CRG cluster A than in CRG cluster B (Fig. 6D). Heatmaps were used to illustrate the different gene expression profiles in the two CRG clusters (Fig. 6E). PCA was then used to verify the correction of the CRG clustering classification (Fig. 6F). A more in-depth analysis of immune cell infiltration was performed considering the association between ALD and the immune microenvironment. As shown in Figs. 6G and 10 types of immune cells showed significant differences between CRG clusters A and B, implying differences in immune responses between the two clusters. The association between immune cell infiltration and the expression profiles of three CRG regulators was confirmed by ssGSEA-based heatmaps (Fig. 6H). In addition, we investigated the relationship between immune cell infiltration and three CRGs by ssGSEA. As shown in Fig. 6I, DPYD was negatively correlated with activated dendritic cells, CD56 bright natural killer cells, CD56dim natural killer cells, and monocytes. SLC31A1 was negatively correlated with immature dendritic cells and positively correlated with monocytes (Fig. 6J). A negative correlation was observed between DBT and activated CD4 T cells, dendritic cells, gamma delta T cells, immature dendrites, MDSCs, macrophages, mast cells, plasmacytoid dendrites, regulatory T cells, T follicular helper cells, and type 1 helper cells (Fig. 6K).

### 3.6 The correlation between the CRG cluster, gene cluster, and CRG score

**Fig. 7:**
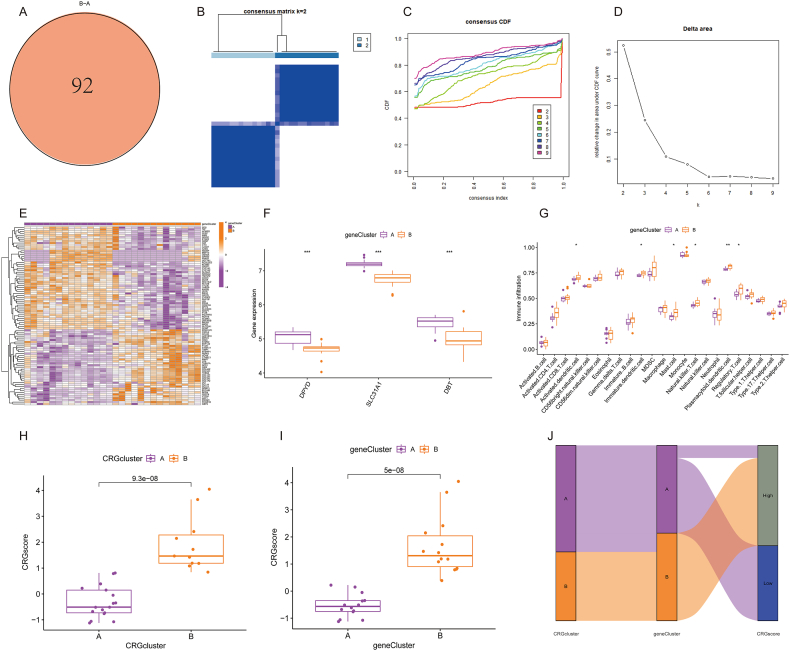
Generation of the CRGs signature and immune microenvironment. (A) 92 DEGs between CRG clusters A and B. (B) Consensus clustering matrix of 92 DEGs for k = 2. (C) The consensus CDF curve based on various k (k = 2–9). (D) Relative increase in cluster stability by delta area fraction. (E) The heatmap showed the distinct expression of CRGs in each gene cluster. (F) The boxplots displayed the expression levels of the three hub CRGs in their respective clusters. (G) Different immune cells in the two CRG clusters. The CRG score was calculated between the A and B groups in distinct CRG clusters (H) and gene clusters (I). (J) A Sankey diagram illustrated the correlations among the CRG clusters, gene clusters, and CRG scores. *p < 0.05, **p < 0.01, ***p < 0.001. DEGs, differential expression genes; CRG, cuproptosis-related gene; CDF, cumulative distribution function.

Ninety-two DEGs overlapping between gene clusters A and B were shown in Fig. 7A. The optimal k value of the gene cluster was set to 2 to ensure compatibility with the number of gene clusters (Fig. 7B–D). Heatmaps were used to show the different expression profiles of CRGs between the two gene clusters (Fig. 7E). Compared with those in gene cluster A, the expression levels of DBT, DPYD, and SLC31A1 were increased in gene cluster B (Fig. 7F). We then identified six types of immune cells in gene cluster B was significantly higher in gene cluster A (Fig. 7G), indicating a significant feature of immune microenvironment infiltration. Box charts were used to observe the differences in CRG scores between CRG clusters and gene clusters. As shown in Fig. 7H, CRG cluster A had a lower CRG score than CRG cluster B. The CRG score in gene cluster A was also lower than that in gene cluster B (Fig. 7I). Therefore, the CRG scores of different CRG clusters and gene clusters differed significantly. The software package “ggalluvial” R was used to construct a Sankey chart to better display the correspondence between CRG scores, CRG clusters, and gene clusters (Fig. 7J).

### 3.7 Identification of drug candidates and ceRNA networks based on marker genes

**Fig. 8:**
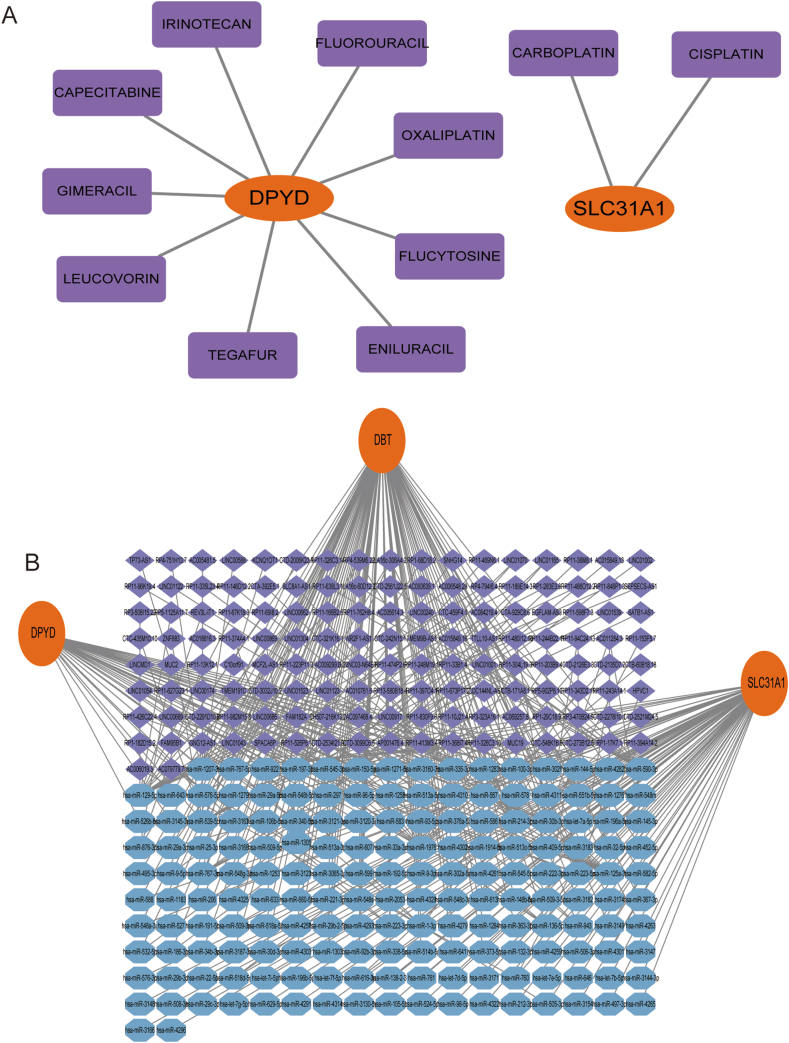
mRNA-drugs and ceRNA network. (A) The purple rectangle nodes symbolize the drugs, while the mRNA-drug interaction network is represented by orange dots. (A) The ceRNA network, based on marker genes, is depicted with blue dots for miRNA and purple dots for lncRNA.

To further explore drug therapy options for ALD, we analyzed the interactions between key genes and drugs by DGIdb. Cytoscape analysis revealed the interaction between genetic markers and drugs (Fig. 8A). A ceRNA network was constructed with the three essential genes using the TargetScan, miRanda, and miRDB databases, revealing 170 miRNAs and 138 lncRNAs (Fig. 8B).

### 3.8 Expression of CRGs in a mouse ALD model and cell alcohol intervention model

**Fig. 9:**
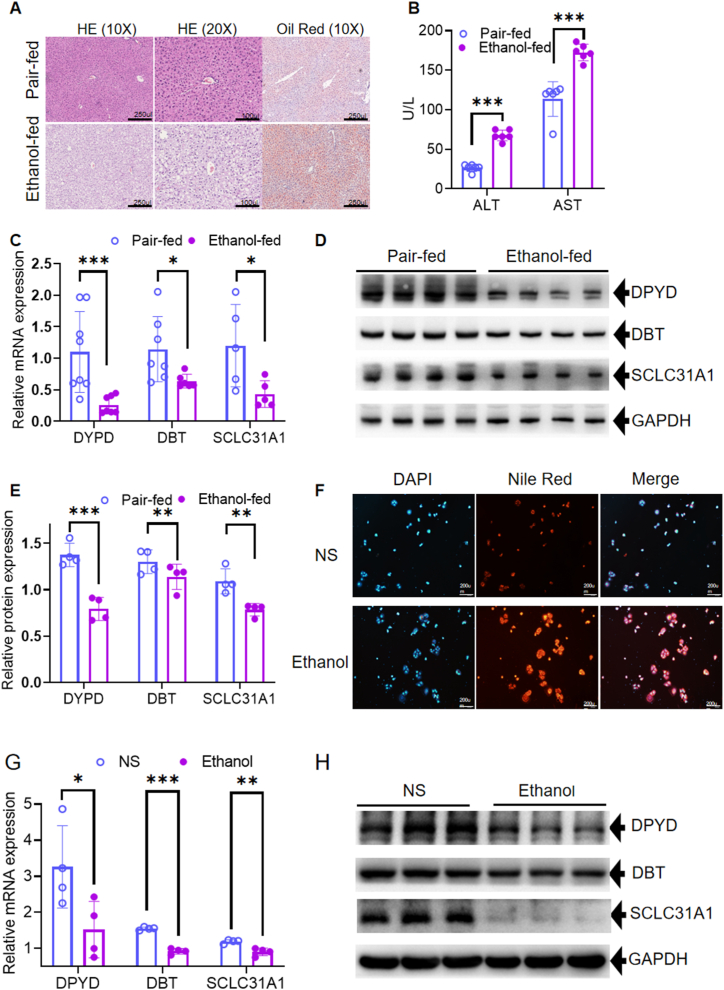
Expression of CRGs in a mouse ALD model and cell alcohol intervention model. The NIAAA mouse ALD model was generated in C57BL/6J mice. Pair-fed mice were used as controls. Serum and liver tissues were collected on day 16 for further analysis. (A) HE and Oil Red O staining. (B) Serum ALT and AST levels. (C) The relative mRNA expression of the three hub genes was verified by qRT‒PCR. (D) The protein expression of the three hub genes was confirmed by Western blotting. (E) The relative protein expression of the three hub genes. (F) Nile red staining. (G) The mRNA levels of the three hub genes in cells with/without ethanol treatment. (H) The protein levels of the three hub genes in cells with/without ethanol treatment. N = 6–8, *p < 0.05, **p < 0.01, ***p < 0.001. CRGs, cuproptosis-related genes; ALD, alcohol-related liver disease; NIAAA, National Institute on Alcohol Abuse and Alcoholism; HE, Hematein & Eosin; ALT, alanine aminotransferase; AST, aspartate aminotransferase; qRT‒PCR, quantitative reverse transcription polymerase chain reaction.

H&E and Oil Red O staining suggested hepatic steatosis in the ALD group (Fig. 9A). AST and ALT levels were significantly higher in the ALD group than in the control group (Fig. 9B). The above results indicate that we successfully established the ALD model. qRT‒PCR measurement of mRNA levels indicated that the expression levels of the three key genes were reduced in the ALD group compared with those in the control group (Fig. 9C). Meanwhile, the expression of the protein amounts of the three genes in ADL and normal liver tissues was significantly different through Western blot (Fig. 9D–E). Nile red staining demonstrated substantial lipid deposition in the cell of the Ethanol group, characterized by the formation of much more fat droplets (Fig. 9F). Taken together, these results collectively indicate the successful establishment of the Cells alcohol intervention model. The mRNA levels of three CRG hub genes were also assessed using qRT-PCR. The results indicated that the expression of DPYD, DBT, and DPYD was significantly down-regulated in the ethanol group compared with the NS group (Fig. 9G). Immunoblot analysis also confirmed a similar decreased expression in the ethanol group (Fig. 9H). These results confirm that these three signature CRGs may play an important regulatory role in ALD development.

## 4 Discussion

Alcohol consumption, particularly when prolonged and intense, can cause alcoholic fatty liver disease, cirrhosis, and even liver cancer. ALD is a major burden worldwide [[38], [39], [40]]. Although many studies have been conducted on ALD, many related mechanisms are not completely clear, and treatment methods are limited. Exploring effective diagnostic biomarkers for ADL and related therapeutic drugs is of substantial clinical value. Cuprotosis is a newly discovered mechanism of copper-dependent cell death that plays an important role in many diseases [21,[41], [42], [43], [44]]. Menkes disease and Wilson disease are two typical genetic diseases caused by mutations in the copper transport ATPase gene [45,46]. Imbalances in the distribution and homeostasis of copper within cells have been linked to neurodegenerative diseases, mitochondrial myopathy, and some types of cancer [47]. In addition, abnormal copper metabolism may also affect the cholesterol biosynthesis pathway, as observed in fish models, where copper exposure leads to significant downregulation of genes in the cholesterol biosynthesis pathway [48]. However, the specific role of cuproptosis in the pathogenesis and regulation of ALD remains unclear. Here, we investigated the possible role of CRGs in ALD, identifying potential key genes and exploring possible target drugs.

We downloaded ALD and control liver data from the GEO database for statistical analysis to identify differentially expressed CRGs and obtained 18 DEGs associated with cuproptosis. These results suggested that CRGs may influence ALD progression. Our correlation analysis showed that the identified DE-CRGs were closely correlated with each other; however, some had no obvious correlation at the protein level. Thus, there was heterogeneity in the interaction of CRGs at the gene and protein levels.

The important role of CRGs in LA metabolism, 2-oxocarboxylic acid metabolism, and ROS production was revealed by GO and KEGG enrichment analyses. LA is an important cofactor of mitochondrial dehydrogenase [49], which is necessary for cell growth, metabolic fuel production, and antioxidant defenses [50]. LA binds well to copper and is also an endogenous membrane permeability metabolite that helps improve copper overload in human diseases [51,52]. LA reduces the toxic effects of copper elevation by altering the cellular redox environment [51]. Copper-dependent cell death occurs through the direct binding of copper to the fatty component of the tricarboxylic acid (TCA) cycle. The accumulation of fatty acylated proteins, resulting in the loss of iron-sulfur (Fe-S) tuftin and the production of protein-toxic stress, culminates in cell death [41]. GO MF analysis showed that the 18 DE-CRGs were significantly correlated with iron-sulfur cluster binding. Previous studies have demonstrated that Fe-S protein is significantly associated with copper deformities [41]. The biological production of cellular iron thionein (Fe/S) is the basic and minimal function of mitochondria [53]. Destabilization and an overall reduction in Fe-S cluster proteins cause proteotoxic stress and mitochondrial dysfunction, and the Fe-S cluster protein FDX1 and lipoylation are key regulators of cuproptosis [41].

LASSO, RF, and SVM-REE analyses of the 18 DE-CRGs revealed three key genes (DPYD, SLC31A1, and DBT) that can effectively predict ALD, with an AUC value of 0.704. Two external datasets were used to verify the validity of the three-gene model, with AUCs of 0.670 and 0.800. The AUC values for the three key genes in both validation datasets exceeded 0.670. The nomogram model, calibration curves, and DCA showed that this model had a good prediction ability and clinical application value. Thus, a predictive model combining these three key genes could serve as a reliable and robust biomarker for the effective prediction of ALD.

DPYD encodes dihydropyrimidine dehydrogenase (DPD), a key enzyme involved in fluoropyrimidine metabolism [54]. DPD is widely distributed in various normal tissues, especially in the liver and peripheral blood mononuclear cells [55]. DPD is the primary and rate-limiting enzyme in the uracil and thymine catabolic pathways [56]. Regulating pyrimidine catabolism protects against the occurrence and development of liver fibrosis [57]. SLC31A1 is a member of the copper transporter family with an extracellular copper ion binding domain and is considered the main transporter of copper ions into the cell [11]. SLC31A1 encodes the high-affinity copper uptake protein CTR1, which is essential for mammalian development and copper homeostasis [58]. Copper overload and deposition are likely related to SLC31A1 [59]. SLC31A1 is abnormally expressed in various cancers and is associated with cancer progression [60]. As a key gene associated with copper metabolism disorder, SLC31A1 is of great value in the prognostic prediction of pancreatic cancer and colorectal cancer [61,62]. The GSEA results indicated that SLC31A1 was involved in extracellular matrix-receptor interactions and O-type glycan biosynthesis. GSVA further showed that high SLC31A1 expression participated in extracellular matrix-receptor interaction and O-type glycan biosynthesis, and low SLC31A1 expression participated in retinol metabolism and steroid hormone biosynthesis. DBT is a subunit of branched-chain alpha-ketoate dehydrogenase and is essential for the second step in branched-chain amino acid catabolism [63,64]. DBT instability can lead to an accumulation of deleterious derivatives [65]. This imbalance in branched-chain amino acid metabolism has been linked to the emergence of ALD [66]. DBT can affect MAPK signaling [63], and regulating MAPK pathways can improve liver disease in mice with ALD [67]. Long-term drinking can cause lipid metabolism disorders and cause liver lesions [66]. Morrell et al. [68] proposed that lipid synthesis was associated with fatty liver disease and copper deficiency. The lipoacyl-binding domain of DBT interacts with annexin A2, activating Hippo signaling, inhibiting tumor progression, and correcting lipid metabolism disorders [69]. The GSEA results indicated that SLC31A1 was involved in amino acid metabolism and extracellular matrix-receptor interactions. ECM proteins affect ROS production [70]. ROS plays an important role in the clinical and pathological profile of ALD by affecting intracellular signaling pathways and disrupting transcriptional control [71].

Cuprotosis has been shown to regulate inflammation [21], and our results indicate that the three CRGs were significantly associated with immune inflammatory cells. Immune cells and inflammation play an important role in ALD; various inflammatory cells play a promoting role, especially interleukin-8 and neutrophils [72]. This study demonstrated a significant distinction between ALD patients and the control group in terms of B cells, CD8 (+) T cells, macrophages, NK cells, T helper cells, Th1 cells, APC coinhibition, CCR, cytolytic activity, accessory inflammatory response, and type II IFN responses. Oxidative stress and inflammation play an important role in ALD [73]. This study showed that DPYD was significantly associated with the type II IFN response, Th1 cells, and NK cells, DBT was significantly associated with MHC class 1, CCR, and aDCs, and SLC31A1 was significantly associated with B cells, APC coinhibition, and CCR. SLC31A1 and DBT were significantly associated with immune cells and immune function [74]. Previous studies have shown that SLC31A1 is significantly associated with immune cells [60,75]. DPYD is associated with immune cells and plays a role in the immune microenvironment [76]. Activated B-cell pathways and macrophages play an important role in ALD [[77], [78], [79]]. Enhanced CD8 (+) T cells mediate alcoholic liver injury [80]. MHC class 1 and NK cells induce hepatocyte apoptosis in ALD [81,82]. It is of substantial clinical significance to explore therapeutic methods for ALD. We conducted gene-targeting drug analysis based on the three key genes identified. Several drugs targeting the DPYD and SLC31A1 genes were antitumor chemotherapy drugs acting on cell biosynthesis. One of these drugs is leucovorin, a derivative of folate, which is a precursor for 5,10-methylene tetrahydrofolate [[83], [84], [85], [86]]. Leucovorin is the coenzyme of a carbon group transferase and is involved in many important reactions, such as purine and pyrimidine synthesis [83]. Folic acid is essential for the normal functioning of organisms. In cells, folic acid is a donor and acceptor of carbon metabolism and is involved in methylation, an important biological process [87]. Folic acid is also involved in the synthesis of nucleic acids, amino acids, and pantothenic acid [88]. Folic acid has been shown to reduce alcohol-induced liver damage, and folic acid supplementation may be useful for the prevention and treatment of ALD [89]. Therefore, leucovorin has the potential to be an effective preventive measure for ALD. Since lncRNAs, miRNAs, and mRNAs interact and affect cellular biosynthesis [[90], [91], [92]], we constructed an mRNA-miRNA-lncRNA regulatory network for ALD, revealing that lncRNAs could regulate the three key genes (DPYD, DBT, and SLC31A1). Therefore, gene-targeted drug analysis provides a new way to search for possible drugs to prevent and treat ALD further, and ceRNA network analysis provides a new avenue for further exploring ALD pathogenesis. These findings require further validation in cell and animal studies.

Based on these findings, therapeutic strategies, such as small interfering RNA (siRNA) or gene editing techniques, are designed and implemented to target genes involved in copper death, with a view to developing new therapies.

Our study has some limitations. First, we performed genetic analysis on data downloaded from the GEO database, and the datasets may have certain biases. Second, the total number of cases was relatively small. Furthermore, we have not yet performed cellular or animal validation of the gene-targeting drugs we discovered.

## 5 Conclusions

We identified three important genes, and by combining these three genes, we can accurately diagnose patients with ALD. Then, we explored the relationship between these genes and invasive immune cells and analyzed the significant heterogeneity in immune responses between ALD patients and control liver samples. Our research unveils the role of copper in ALD, providing a new theoretical foundation for the potential pathogenesis of ALD and therapeutic options.

## Ethics approval and consent to participate

All animal experiments were approved by the Animal Care and Use Committee of the First Affiliated Hosputal of Guangxi Medical University (NO.2023-S606-01).

## Consent for publication

Not applicable.

## Data availability

The datasets in this study were enrolled from the GEO database (https://www.ncbi.nlm.nih.gov/geo/), with the following data accessions enrolled: GSE28619, GSE103580, GSE142530 and GSE155907. This data can be found here: https://www.jianguoyun.com/p/DT-bA08QjrmsDBjJ-bIFIAA.

## Funding

This work was supported in part by the 10.13039/501100012166National Key Research and Development Program (2022YFE0131600); Guangxi Science and Technology Base and Talent Project (GuikeAA21220002); The 10.13039/501100013314111 Project (D17011); Advanced Innovation Teams and Xinghu Scholars Program of 10.13039/501100011827Guangxi Medical University.

## CRediT authorship contribution statement

**Jiangfa Li:** Writing – review & editing, Writing – original draft, Validation, Methodology, Formal analysis, Data curation. **Yong Wang:** Writing – original draft, Methodology, Formal analysis, Data curation. **Zhan Wu:** Methodology, Investigation. **Mingbei Zhong:** Methodology, Investigation. **Gangping Feng:** Methodology, Investigation, Formal analysis. **Zhipeng Liu:** Validation, Methodology. **Yonglian Zeng:** Supervision, Software, Data curation. **Zaiwa Wei:** Methodology, Formal analysis. **Sebastian Mueller:** Methodology, Investigation, Data curation. **Songqing He:** Visualization, Validation, Methodology, Investigation, Funding acquisition, Conceptualization. **Guoqing Ouyang:** Supervision, Software, Resources, Methodology, Investigation, Formal analysis, Data curation. **Guandou Yuan:** Writing – review & editing, Visualization, Supervision, Methodology, Investigation, Funding acquisition, Formal analysis, Data curation, Conceptualization.

## Declaration of competing interest

The authors declare that they have no known competing financial interests or personal relationships that could have appeared to influence the work reported in this paper.
