# Supplementary material for: Identification of diagnostic markers and molecular clusters of cuproptosis-related genes in alcohol-related liver disease based on machine learning and experimental validation
Source: Heliyon. 2024 Sep 12;10(18):e37612. doi: 10.1016/j.heliyon.2024.e37612 (PMC11417179; doi:10.1016/j.heliyon.2024.e37612)
Supplement: Multimedia component 1 [file mmc1.docx]

**Identification of diagnostic markers and molecular clusters of cuproptosis-related genes in alcohol-related liver disease based on machine learning and experimental validation**

Jiangfa Li^1,2,3,#^, Yong Wang^1,2,3,#^, Zhan Wu^1,2,3^, Mingbei Zhong^1,2,3^, Gangping Feng^1,2,3^, Zhipeng Liu^1,2,3,^, Yonglian Zeng^1,2,3,^, Zaiwa Wei^1,2,3^, Sebastian Mueller^4^, Songqing He^1,2,3^*, Guoqing Ouyang^1,2,3^*, [Guandou Yuan](https://pubmed-ncbi-nlm-nih-gov-443--bjmu.jitui.me/?term=Yuan+G&cauthor_id=35069557)^1,2,3^*

^1^Division of Hepatobiliary Surgery, The First Affiliated Hospital of Guangxi Medical University, Nanning, Guangxi 530021, China.

^2^Key Laboratory of Early Prevention and Treatment for Regional High Frequency Tumor (Guangxi Medical University), Ministry of Education, Nanning, Guangxi 530021, China.

^3^Guangxi Key Laboratory of Immunology and Metabolism for Liver Diseases, Nanning, Guangxi 530021, China.

^4^Center for Alcohol Research, University Hospital Heidelberg, Heidelberg, Germany.

^#^ These authors have contributed equally to this work

*Correspondence to:

Guandou Yuan, Division of Hepatobiliary Surgery, The First Affiliated Hospital of Guangxi Medical University, NO 6 Shuangyong Road, Nanning 530021, Guangxi, China. E-mail: [dr_yuangd@gxmu.edu.cn](mailto:dr_yuangd@gxmu.edu.cn)

Guoqing Ouyang, Division of Hepatobiliary Surgery, The First Affiliated Hospital of Guangxi Medical University, NO 6 Shuangyong Road, Nanning 530021, Guangxi, China. Email: [Ouyangguoqing@stu.gxmu.edu.cn](mailto:Ouyangguoqing@stu.gxmu.edu.cn).

Songqing He, Division of Hepatobiliary Surgery, The First Affiliated Hospital of Guangxi Medical University, NO 6 Shuangyong Road, Nanning 530021, Guangxi, China. Email: [dr_hesongqing@163.com](mailto:dr_hesongqing@163.com)

**Contents**

[Supplementary Figures 2](#_Toc18502)

[Figure S1 3](#_Toc15950)

[Figure S2 3](#_Toc14488)

[Figure S3 4](#_Toc14153)

[Figure S4 5](#_Toc10876)

[Figure S5 6](#_Toc6970)

[Figure S6 7](#_Toc24939)

[Figure S7 8](#_Toc8008)

[Figure S8 9](#_Toc26449)

[Figure S9 10](#_Toc12678)

[Table S1 List of Primers 11](#_Toc13929)

**Supplementary Figures**

**Figure S1**

**
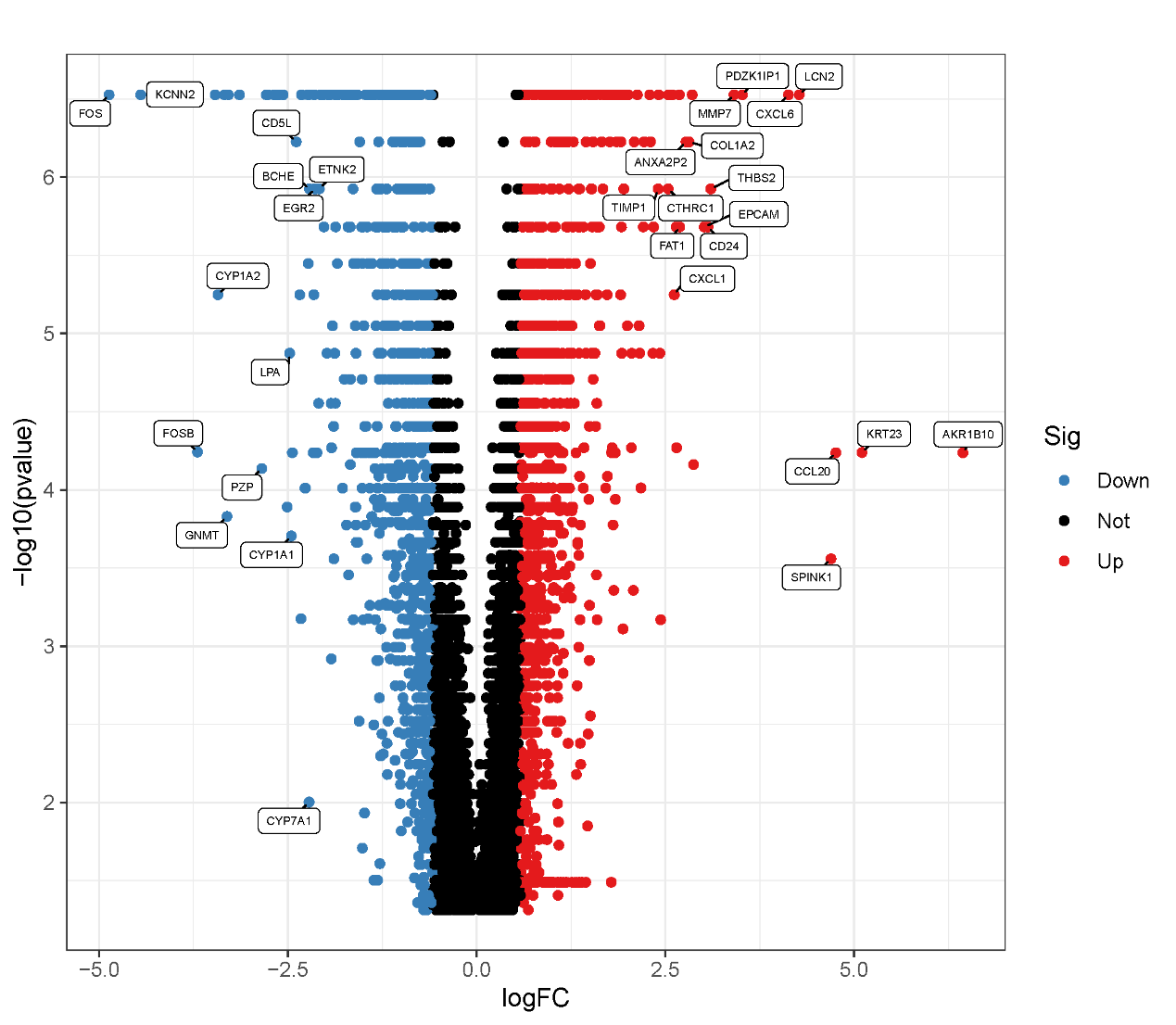
**

**Volcano plot of the differentially expressed genes (DEGs).**

The over-expressed genes are marked in red and the down-expressed genes are marked in blue.

**Figure S2**


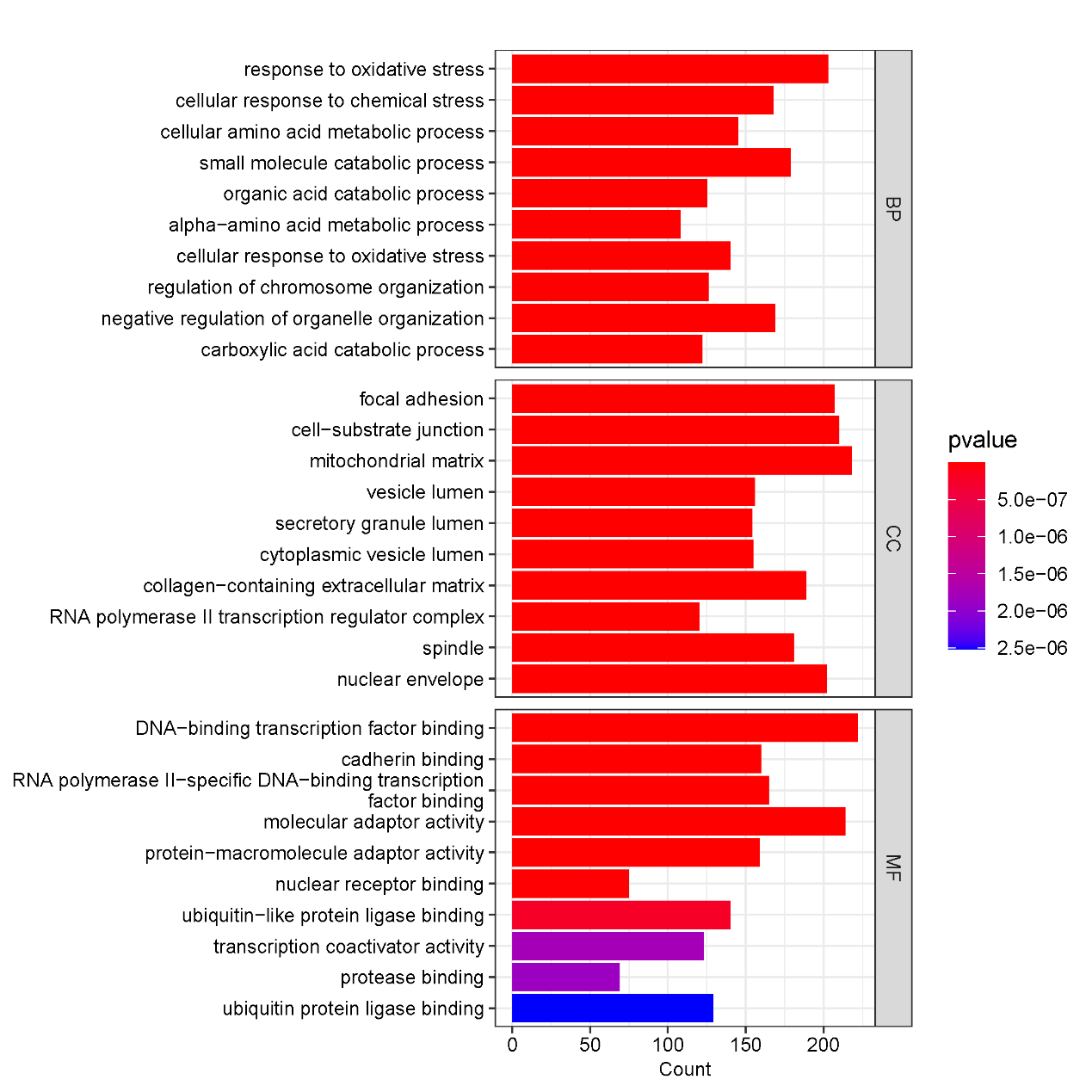


**Gene ontology (GO) analysis of the differentially expressed genes (DEGs).**

**Figure S3**

**
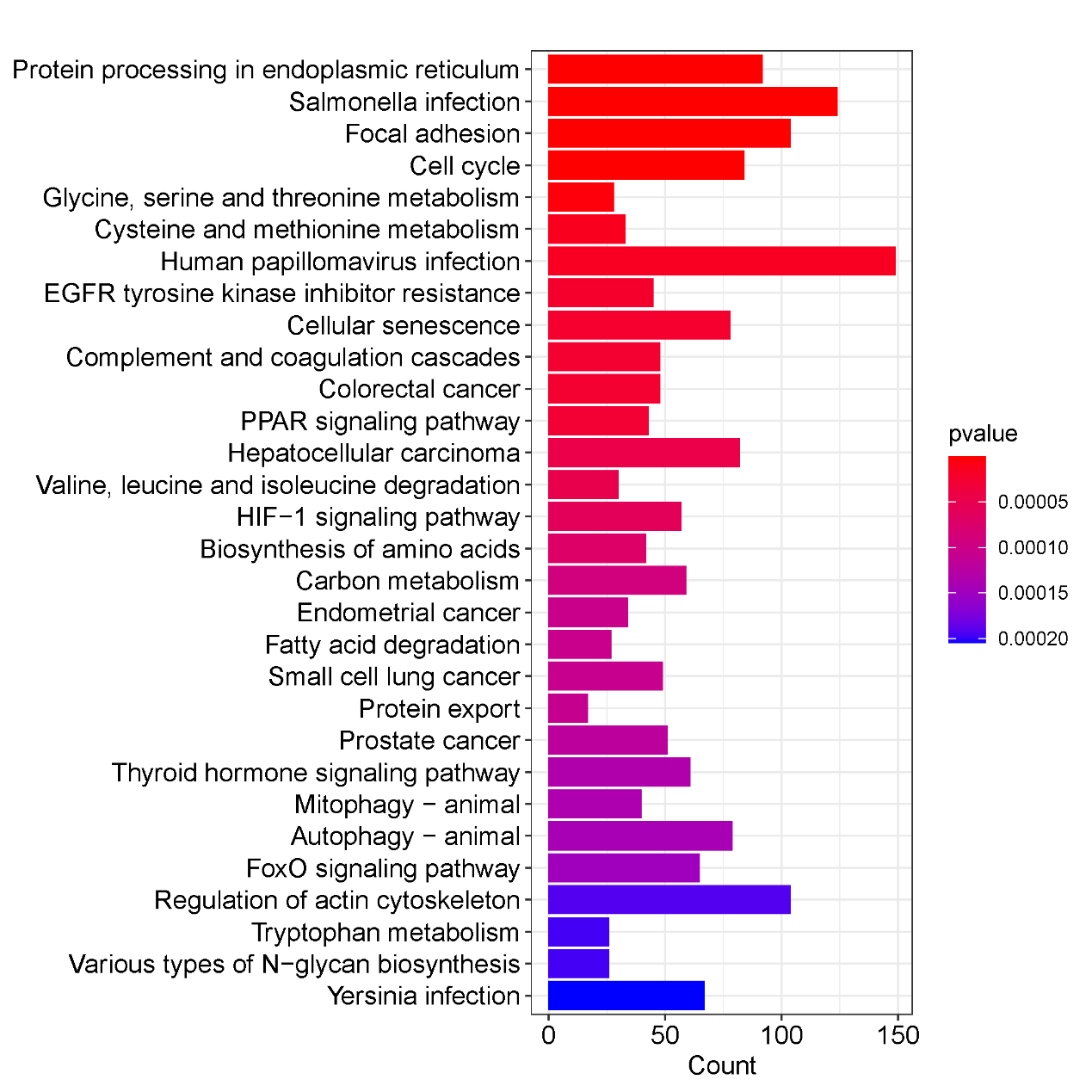
**

**KEGG analysis of the differentially expressed genes (DEGs).**

**Figure S4**


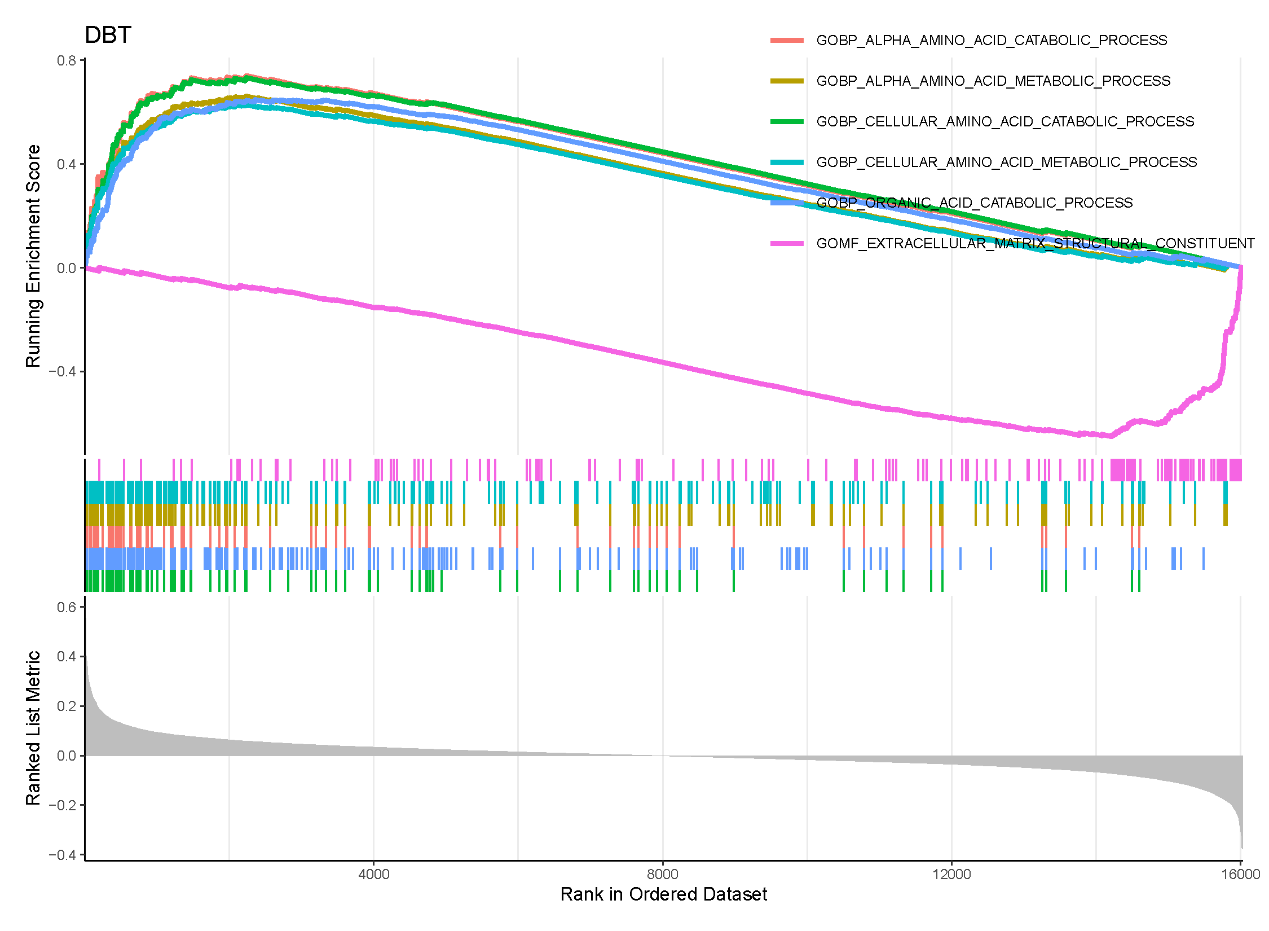


The GO pathway enrichment analysis of DBT were carried out by GSEA enrichment method, and the two items with the highest and lowest enrichment scores are visualized according to the arrangement of enrichment scores.

**Figure S5**

**
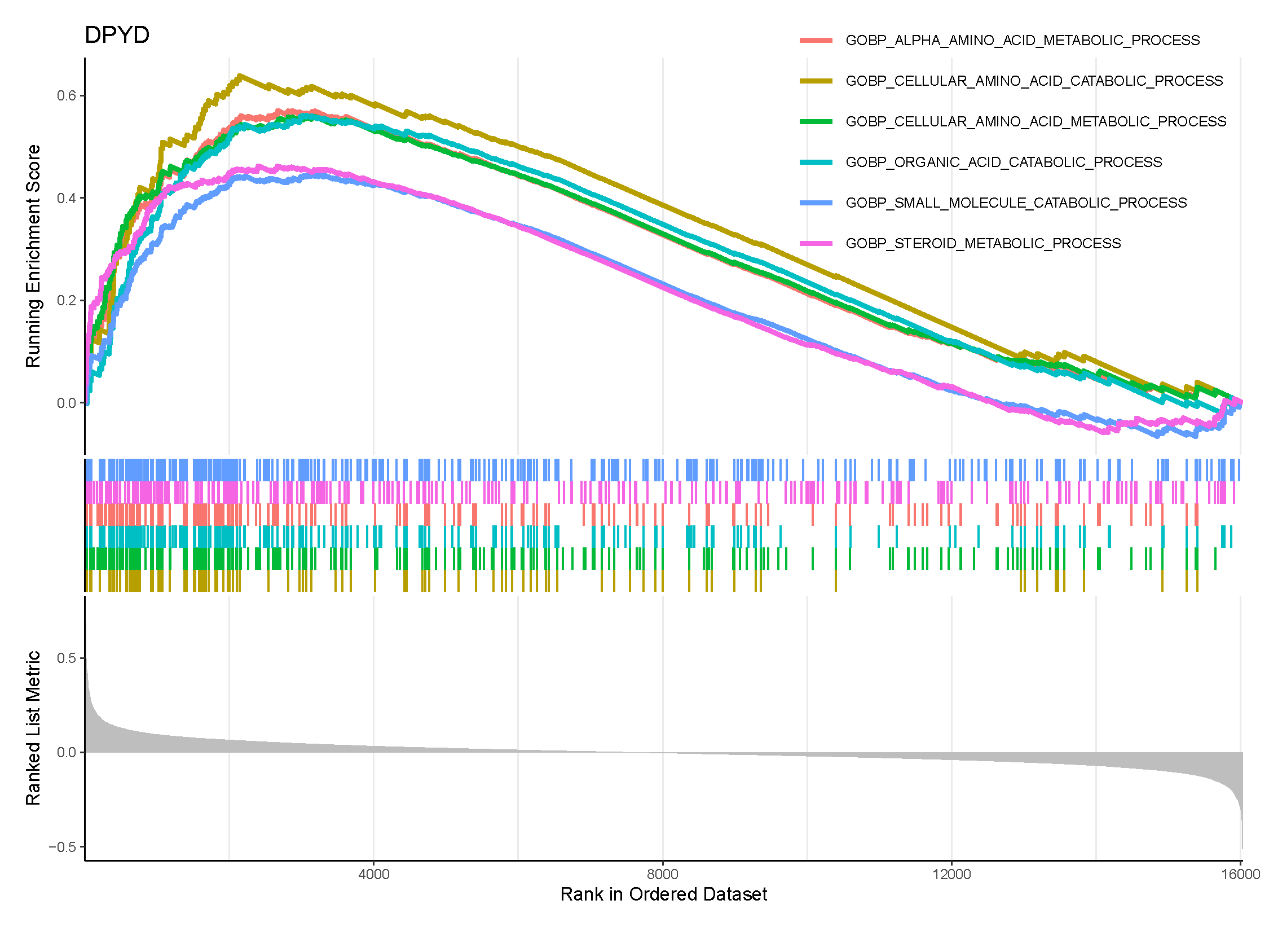
**

The GO pathway enrichment analysis of DPYD were carried out by GSEA enrichment method, and the two items with the highest and lowest enrichment scores are visualized according to the arrangement of enrichment scores.

**Figure S6**


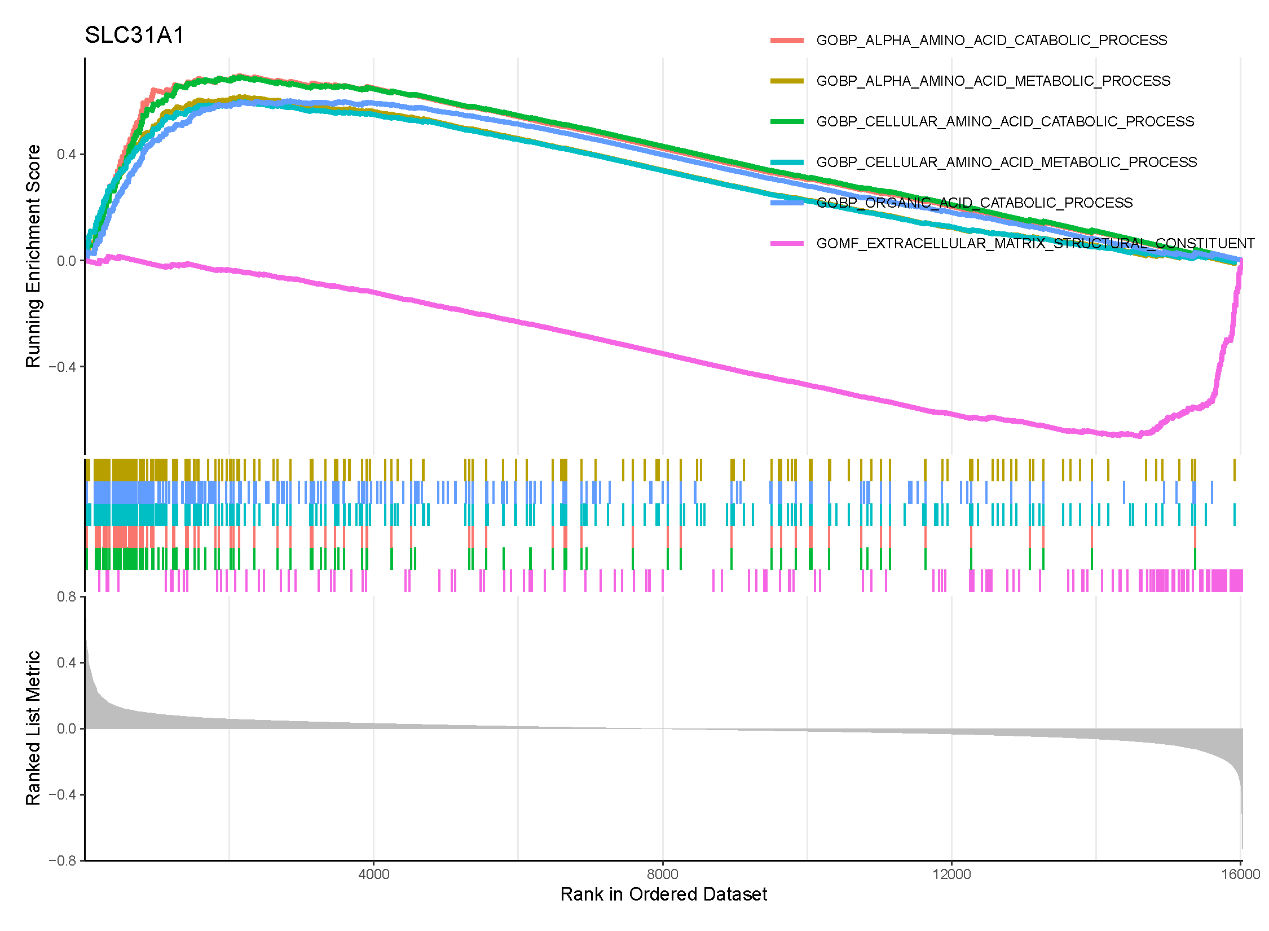


The GO pathway enrichment analysis of SLC31A1 were carried out by GSEA enrichment method, and the two items with the highest and lowest enrichment scores are visualized according to the arrangement of enrichment scores.

**Figure S7**


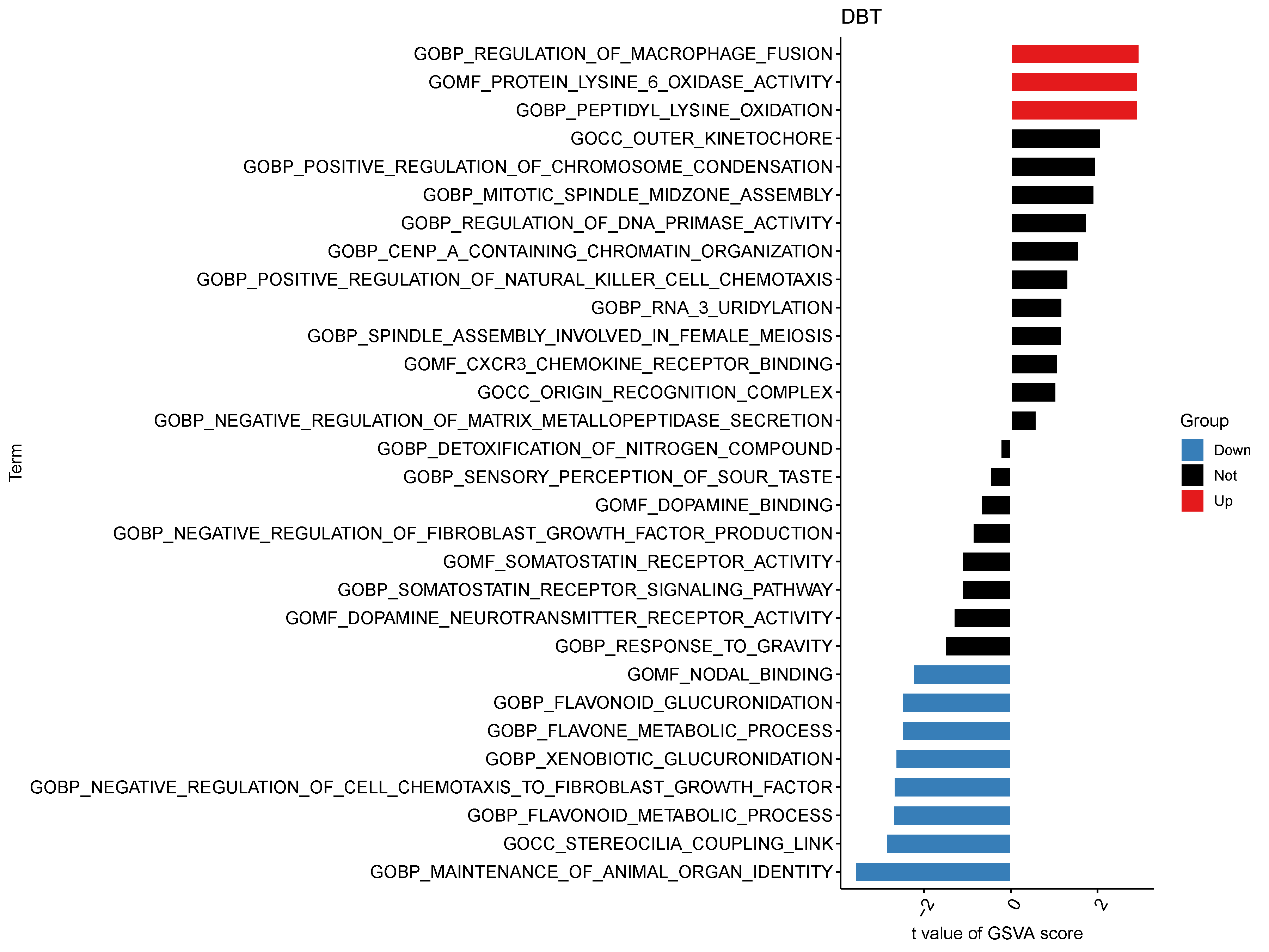


The GO pathway enrichment analysis of DBT was carried out by GSVA enrichment method, and the top 50 are visualized according to the enrichment score. The over-expressed genes are marked in red and the down-expressed genes are marked in blue.

**Figure S8**


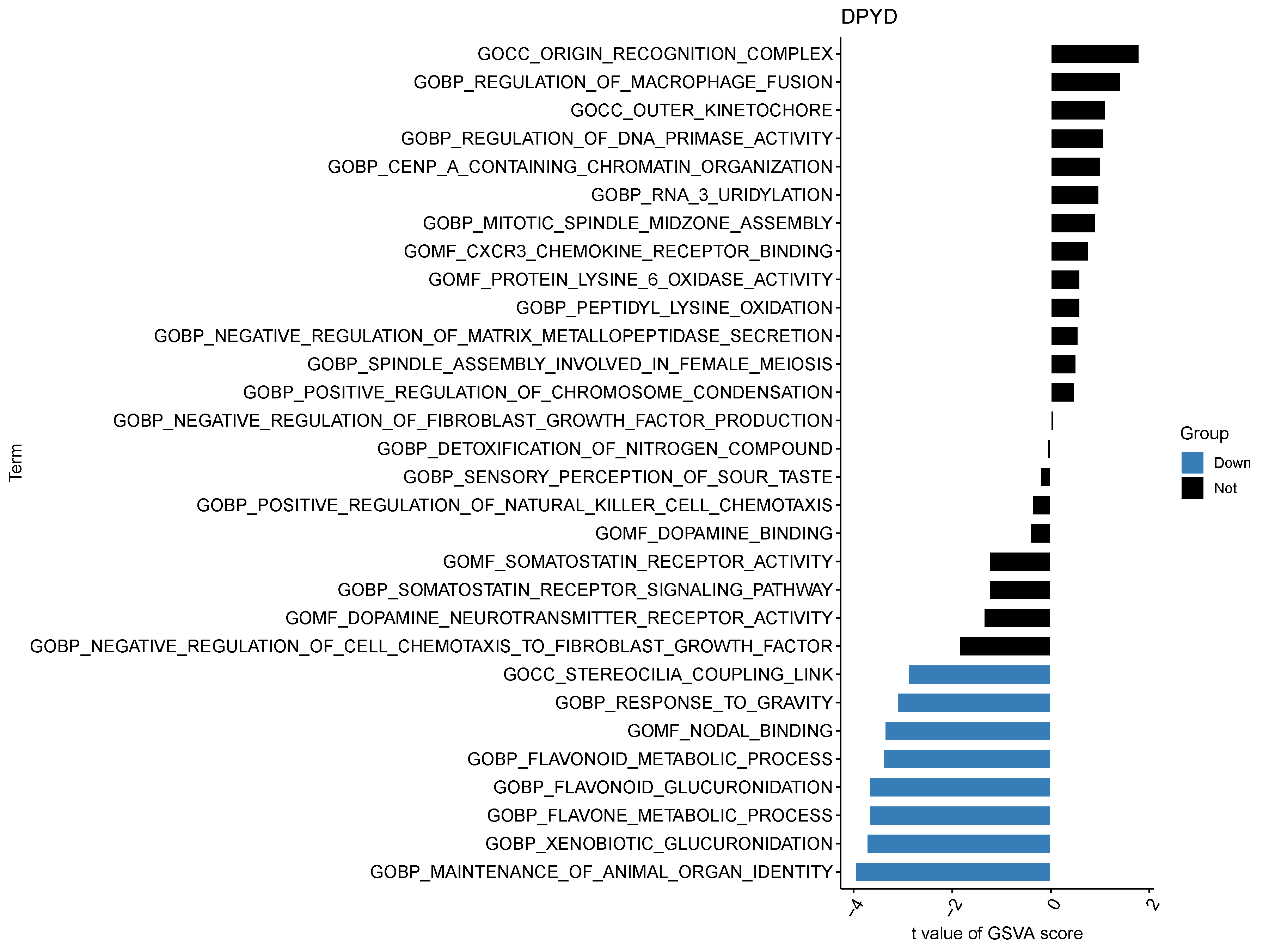


The GO pathway enrichment analysis of DPYD was carried out by GSVA enrichment method, and the top 50 are visualized according to the enrichment score. The the down-expressed genes are marked in blue.

**Figure S9**


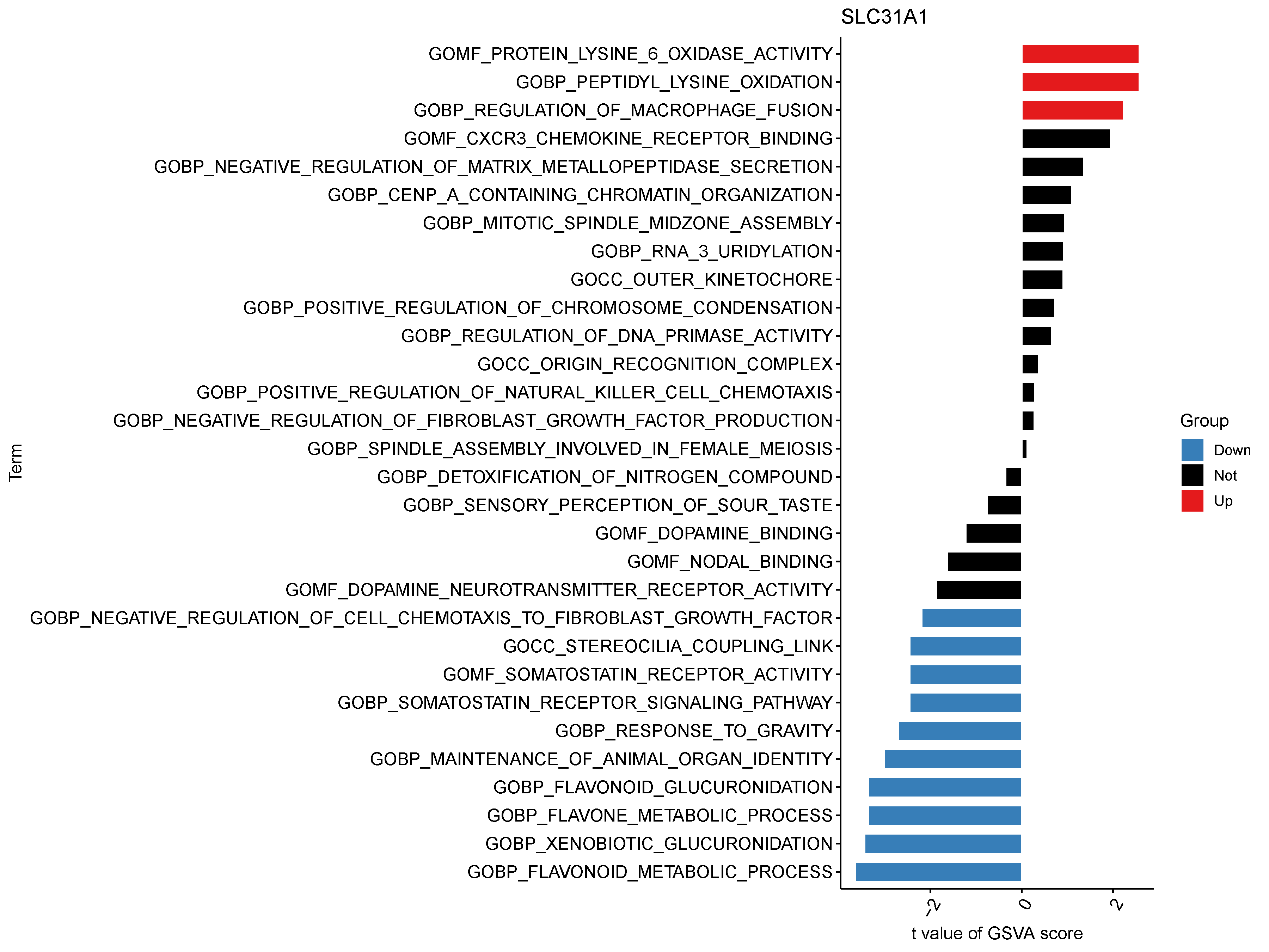


The GO pathway enrichment analysis of SLC31A1 was carried out by GSVA enrichment method, and the top 50 are visualized according to the enrichment score. The over-expressed genes are marked in red and the down-expressed genes are marked in blue.

**Table S1 List of Primers**

| **Primer name** | | **Primer sequence(5'to3')** |
| --- | --- | --- |
| Dpyd | Forward | GAGTACAAGCTCATGCAACTCT |
|  | Reverse | GCTTCTCACAGGTAAAGCAGT |
| Slc31a1 | Forward | TATGAACCACACGGACGACAA |
|  | Reverse | GCCATTTCTCCAGGTGTATTGA |
| Dbt | Forward | GAGATACGGTGTCTCAGTTTGAC |
|  | Reverse | GCCTCTTAATGACGCCATCGTAA |
