# Supplementary material for: Identification of diagnostic markers and molecular clusters of cuproptosis-related genes in alcohol-related liver disease based on machine learning and experimental validation
Source: Heliyon. 2024 Sep 12;10(18):e37612. doi: 10.1016/j.heliyon.2024.e37612 (PMC11417179; doi:10.1016/j.heliyon.2024.e37612)
Supplement: Multimedia component 2 [file mmc2.pdf]

AW: Cuproptosis in ALD

发件人：Sebastian\x26nbsp;Mueller<sebastian.mueller@urz.uni-heidelberg.de>
时　间：2023年12月10日(星期天) 凌晨2:06
收件人：袁观斗<dr_yuangd@gxmu.edu.cn>

Dear Guandou,

Many thanks for the nice manuscript that I had the chance to briefly read during this week despite limited time. In general, the data are nice and timely and the paper is well structured and written.

Where necessary, I made a few comments or corrected some typos. Below are some general comments that you may want to consider. I tried to finish the reading today in case you want to briefly discuss it tomorrow during our meeting.

Best regards

Sebastian

POINTS:

Just terminology: ALD is now more generally termed alcohol-related liver disease.
The introduction looks sometimes redundant but could be limited to the essentials. Wilson's disease could be also mentioned. In fact, in copper-overload Wilson disease, a relation between iron and copper metabolism has been noted early on.
I miss a little bit in this paper the patient characteristics. How much alcohol did they consume. This information would be important to have a better idea about the study cohort. Likewise, it would be good to have an impression about the control cohort? Normal laboratory values or histology do not exclude ALD nor alcohol consumption. Here, the animal study is more clear.
Just for inspiration or you may consider to integrate in the discussion or intro section: We regularly induce eryptosis in red blood cells using copper sulfate to prime them for erythrophagocytosis or efferocytosis. As our mortality study has identified for the first time enhanced red blood cell turnover as most important progression factor in ALD, this may be of high relevance in cuproptosis and ALD. Here is a text that you modify for inclusion with the references:

Text:

Hemolytic anemia, hemolysis and enhanced red blood cell turnover has been recently identified as key prognostic factor for the survival of chronic heavy drinkers (1). One mechanism of enhanced red blood cell turnover in ALD is the ingestion of red blood cells by macrophages (erythrophagocytosis) and hepatocytes (efferocytosis) (2, 3). Both erythrophagocytosis and efferocytosis can also be initiated by treatment of red blood cells with copper sulfate most likely through induction of eryptosis or oxidation of the RBC cellular membrane (2, 3) thus linking copper to cell death.

You may also quote the newly released book on ALD (4)

References

1. Mueller S, Mueller J. Alcohol and mortality: first preliminary lessons from a prospective 15 year follow-up study. In: Mueller S, Heilig M, editors. Alcohol and Alcohol-related Diseases: Springer International Publishing Cham; 2023. p. 81-102.

2. Mueller S, Mueller J, Li S, Zheng C, Chen C. Hepatic Iron Overload in Heavy Drinkers: Molecular Mechanisms and Relation to Hemolysis and Enhanced Red Blood Cell Turnover. In: Mueller S, Heilig M, editors. Alcohol and Alcohol-related Diseases: Springer; 2023. p. 1075-106.

3. Mueller S, Chen C, Mueller J, Wang S. Novel Insights into Alcoholic Liver Disease: Iron Overload, Iron Sensing and Hemolysis. J Transl Int Med. 2022;10(2):92-124.

4. Mueller S, Heilig M. Alcohol and Alcohol-related Diseases: Springer Cham; 2023.

Von: 袁观斗 <dr_yuangd@gxmu.edu.cn>
Gesendet: Montag, 4. Dezember 2023 04:39
An: sebastian.mueller <sebastian.mueller@urz.uni-heidelberg.de>
Betreff: Cuproptosis in ALD

Dear Prof. Mueller,

Just like I mentioned in my last email, we prepared a manuscript entitled "**Identification of diagnostic markers and molecular clusters of cuproptosis-related genes in alcoholic liver disease based on machine learning and experiment validation**", which would like to try Cellular & Molecular Biology Letters first. We would like to list you as a co-author, which will be better for our further cooperation. Could you please spare some time to review this manuscript? We appreciate your kind help.

Looking forward to hearing from you.

Best,

Guandou
